# Pathogenic *Leishmania* spp. detected in lizards from Northwest China using molecular methods

**DOI:** 10.1186/s12917-019-2174-4

**Published:** 2019-12-09

**Authors:** Jun-Rong Zhang, Xian-Guang Guo, Han Chen, Jin-Long Liu, Xiong Gong, Da-Li Chen, Jian-Ping Chen

**Affiliations:** 1 Department of Parasitology, West China School of Basic Medical Sciences and Forensic Medicine, Sichuan University, Chengdu, 610041 China; 2 Chengdu Institute of Biology, Chinese Academy of Sciences, Chengdu, 610041 China; 3 University of Chinese Academy of Sciences, Beijing, 100039 China

**Keywords:** Lizard, *Leishmania*, Pathogenic, Mixed infection, Haplotypes, Northwest China

## Abstract

**Background:**

Leishmaniosis, a disease caused by pathogenic *Leishmania* parasites, remains an unresolved health problem in the New World and the Old World. It is well known that lizards can be infected by a subgenus of *Leishmania* parasites, i.e. *Sauroleishmania*, which is non-pathogenic to humans. However, evidence suggests that lizards may also harbor pathogenic *Leishmania* species including the undetermined *Leishmania sp*., discovered in our previous work. *Leishmania* DNA in lizard blood can be detected by using molecular methods, such as the polymerase chain reaction (PCR).

**Results:**

Three hundred and sixteen lizards, representing 13 species of four genera, were captured for blood samples collection in Northwest China. Two reliable molecular markers (cytochrome *b* and heat shock protein 70 genes) were used for detection in the lizard blood samples, to confirm a widespread presence of pathogenic *Leishmania* parasites and the distribution pattern of *Leishmania* spp. in lizards from Northwest China. The PCR data indicated positive detection rate for *Leishmania* in all the tested lizards with an overall prevalence of 57.91% (183/316). Apart from lizard parasites like *Leishmania tarentolae* and *Leishmania sp*., several pathogenic *Leishmania* including *L. turanica*, *L. tropica* and *L. donovani* complex were identified by using phylogenetic analysis. Co-existence of different haplotypes was observed in most *Leishmania* DNA-positive lizards with an overall rate of 77.6% (142/183). Even mixed infections with different *Leishmania* species appeared to occur in the lizards with an overall rate of 37.7% (69/183).

**Conclusions:**

Lizards can harbor pathogenic *Leishmania* spp. Co-existence of different haplotypes or even species of *Leishmania* indicates mixed infections in natural lizard host. Lizards may contribute to the spread of *Leishmania* parasites. The pathogenic *Leishmania* species detected in lizards from Northwest China may be of great eco-epidemiological importance.

## Background

With over 6500 species, lizards are a widespread group of squamate reptiles that occur across all continents except Antarctica [1]. Lizards are known reservoirs for numerous parasites, such as kinetoplastea and apicomplexan parasites including coccodea and haematozoea [2–6] and thus play important roles in ecological processes. Several investigations have reported the isolation of a subgenus of *Leishmania* from lizards, i.e. *Sauroleishmania* [7–9]. Currently, it is widely accepted that *Leishmania* strains from reptiles are non-pathogenic for warm-blooded organisms [7, 8]; however, doubt rises that lizards may harbor human pathogens, with several reports on amastigotes described within lizard blood cells [10, 11]. The role of reptiles in the epidemiology of leishmaniosis is still controversial and is far from being completely understood [7, 12].

Human leishmaniosis is endemic in 97 countries and territories around the world, and remains a major public health problem. Caused by different species of the genus *Leishmania*, the condition manifests from self-healing cutaneous leishmaniosis (CL) to muco-cutaneous leishmanosis (MCL) and fatal visceral leishmaniosis (VL) [13]. In China, leishmaniosis is still an important public health problem and VL is the main form. Around 1951, 530,000 cases in more than 650 counties were reported. Although leishmaniosis has been eliminated in most areas after powerful national control measures, it remains endemic in the western region of China, especially in Xinjiang Uygur Autonomous Region, Gansu, and Sichuan provinces [14]. According to the epidemiological characteristics, VL is divided into three forms in China: the anthroponotic type (AVL), the zoonotic mountain type (MT-ZVL), and the zoonotic desert type (DT-ZVL) [15]. Four different sand fly species have been verified as vectors: *Phlebotomus chinensis*, *P. longiductus*, *P. wui*, and *P. alexandri* [16]. DT-ZVL is largely distributed in the northwestern region of China where ancient oases and deserts are closely located nearby, no reservoir host has yet been identified [17]. Two recent outbreaks of DT-ZVL, reported in Xinjiang, have called for effective control and prevention in this region [18, 19]. While dogs are recognized as particularly important reservoirs, few dogs and other canids can be found in these areas. Although there are some reports of wild and domestic animals infected with *Leishmania* [20–22], no reservoir host has been identified in this region.

Recent reports of domestic animals infected with *Leishmania* in this region suggest that there may be other potential reservoirs. A previous study has established a method of screening lizards for *Leishmania* infections and it has shown to be of great value for research [23]. In the previous study three species of *Leishmania* were found to exist in lizards, including two pathogenic *Leishmania* species, i.e. *L. tropica* and *L. donovani*. This supports the idea that reptiles should be considered as potential hosts for *Leishmania* infections [23]. In order to confirm whether there is a widespread presence of more pathogenic *Leishmania* parasites and to test the pattern of low host specificity of *Leishmania* spp. in lizards from Northwest China, more reliable molecular markers and samples should be used for detection.

## Results

### Lizards sampling and *Leishmania* detection

A total of 316 blood samples from lizards representing 13 species were obtained from 31 points in Northwest China (Table 1). The species of lizard, captured in each location, range from one to four. The number of lizards, captured for each species, ranged from six (*Trapelus sanguinolentus*) to 83 (*Phrynocephalus versicolor*). Two gene markers were used to genotype the DNA obtained from the blood samples of lizards in this study: Cyt *b* and HSP70. One hundred and eighty-three samples were observed to be positive for *Leishmania* DNA by PCR, indicating a high prevalence of *Leishmania* infection at 57.91% (95% CI, 52.47–63.35%).

**Table 1:** Species and numbers of lizards collected in different districts

District	Site	Point number	Lizard species	Number of lizards
Bortala Mongolia Autonomous Canton, Xinjiang Uygur Autonomous Region	S1: Jinghe County	Point 1	*Phrynocephalus melanurus*	24
			*Eremias velox*	2
Ili Kazak Autonomous Prefecture, Xinjiang Uygur Autonomous Region	S2: Xinyuan County	Point 2	*Eremias arguta*	4
			*Lacerta agilis*	4
	S3: Gongliu County	Point 3	*Eremias velox*	3
		Point 4	*Eremias arguta*	16
		Point 5	*Eremias velox*	11
	S4: Nilke County	Point 6	*Eremias arguta*	1
		Point 7	*Eremias arguta*	10
			*Lacerta agilis*	1
	S5: Tekes County	Point 8	*Eremias arguta*	4
			*Lacerta agilis*	1
	S6: Zhaosu County	Point 9	*Lacerta agilis*	1
	S7: Huocheng County	Point 10	*Eremias velox*	2
			*Trapelus sanguinolentus*	2
		Point 11	*Eremias velox*	8
			*Eremias grammica*	9
			*Trapelus sanguinolentus*	4
			*Phrynocephalus alpherakii*	11
Changji hui Autonomous Prefecture, Xinjiang Uygur Autonomous Region	S8: Fukang City	Point 12	*Phrynocephalus melanurus*	3
			*Eremias velox*	1
	S9: Qitai County	Point 13	*Eremias multiocellata*	10
		Point 14	*Phrynocephalus grumgrzimailoi*	4
	S10: Mori Kazakh Autonomous County/ Mori County	Point 15	*Eremias multiocellata*	12
			*Phrynocephalus grumgrzimailoi*	36
Hami Prefecture, Xinjiang Uygur Autonomous Region	S11: Barkol Kazakh Autonomous County/ Barkol County	Point 16	*Phrynocephalus versicolor*	8
		Point 17	*Eremias vermiculata*	9
			*Phrynocephalus versicolor*	3
		Point 18	*Phrynocephalus versicolor*	7
		Point 19	*Phrynocephalus versicolor*	2
		Point 20	*Phrynocephalus versicolor*	1
	S12: Yiwu County	Point 21	*Phrynocephalus versicolor*	15
		Point 22	*Phrynocephalus versicolor*	2
			*Eremias vermiculata*	3
		Point 23	*Phrynocephalus versicolor*	18
		Point 24	*Phrynocephalus versicolor*	1
		Point 25	*Phrynocephalus versicolor*	20
	S13: Hami City	Point 26	*Eremias vermiculata*	3
			*Phrynocephalus versicolor*	3
			*Phrynocephalus axillaris*	3
		Point 27	*Phrynocephalus axillaris*	1
		Point 28	*Phrynocephalus versicolor*	3
			*Phrynocephalus axillaris*	3
Urumqi City, Xinjiang Uygur Autonomous Region	S14: Dabancheng district	Point 29	*Eremias velox*	4
			*Phrynocephalus axillaris*	5
		Point 30	*Eremias velox*	3
			*Phrynocephalus grumgrzimailoi*	7
Jiuquan City, Gansu Province	S15: Akesai Kazakh Autonomous County/Akesai County	Point 31	*Phrynocephalus vlangalii*	8

As shown in Table 2, all examined species of lizards were found to be positive for *Leishmania* and the prevalence of *Leishmania* DNA in different species of lizards was significant different (*P* = 0.00, < 0.05). The prevalence of *Leishmania* infection in *Phrynocephalus alpherakii* was 90.91% (95% CI, 73.92–107.9%), which was much higher than that in any other species. This was especially significant compared to that in *Lacerta agilis*, which was 16.67% (95% CI, 13.15–46.49%) (*P* = 0.02, < 0.05). The prevalence of *Leishmania* infection in *P. melanurus* and *P. axillaris* was 77.78% (95% CI, 62.1–93.46%) and 83.33% (95% CI, 62.24–104.42%), respectively. For *P. versicolor*, a total of 83 lizards were captured from 12 districts with an infection rate  of 72.29% (95% CI, 62.66–81.92%).

**Table 2:** *Leishmania* DNA detection in blood samples from different lizard species

Lizard species	Number of lizards	Number of positive lizards	Prevalence	95% CI	Number of positive lizards (Infected by)							
					LS	LD	LT	LS + LD	LS + LT	LT + LD	LT + LS + LD	LTR + LS + LD
*Phrynocephalus melalurus*	27	21	77.78%	62.10–93.46%	21	0	0	9	–	–	–	1
*Eremias velox*	34	16	47.06%	30.28–63.84%	8	11	4	3	3	2	1	–
*Eremias arguta*	36	20	55.56%	39.33–71.79%	10	10	1	1	–	–	–	–
*Lacerta agilis*	6	1	16.67%	−13.15–46.49%	1	1	0	1	–	–	–	–
*Trapelus sanguinolentus*	6	2	33.33%	−4.39–71.05%	1	2	0	1	–	–	–	–
*Eremias grammica*	9	3	33.33%	2.53%-–64.13%	0	3	0	–	–	–	–	–
*Phrynocephalus alpherakii*	11	10	90.91%	73.92–107.90%	4	10	0	4	–	–	–	–
*Eremias multiocellata*	22	10	45.45%	24.64–66.26%	2	10	0	2	–	–	–	–
*Phrynocephalus grumgrzimailoi*	47	13	27.66%	14.87–40.45%	3	12	2	2	1	1	–	–
*Phrynocephalus versicolor*	83	62	72.29%	62.66–81.92%	31	53	12	23	9	6	4	–
*Eremias vermiculata*	15	10	66.67%	42.81–90.53%	3	10	0	3	–	–	–	–
*Phrynocephalus axillaris*	12	10	83.33%	62.24–104.42%	5	9	1	5	–	–	–	–
*Phrynocephalus vlangalii*	8	5	62.5%	28.95–96.05%	3	5	2	3	2	2	2	–
Total	316	183	57.91%	52.47–63.35%	92	145	22	57	15	11	7	1

*LS Leishmania sp.*, *LT Leishmania turanica*, *LD Leishmania donovani* complex, *LTR Leishmania tropica*

The 31 points belong to 15 districts located in six regions and two provinces, where sandflies were reported and DT-ZVL is endemic in these points. As shown in Table 3, infection with *Leishmania* DNA in lizards from different locations were also significant different (*P* = 0.00, < 0.05). Absence of *Leishmania* DNA from lizards in Zhaosu County and Fukang County may be related to the small number of the samples (only one and four lizards, respectively) (Table 3). In Hami City, all the sampling lizards were infected by *Leishmania*. The prevalence of *Leishmania* infection in Jinghe County, Yiwu County and Dabancheng district was 88.46% (95% CI, 76.18–100.74%), 76.27% (95% CI, 65.41–87.13%) 78.95% (95% CI, 60.62–97.28%), respectively. Fourteen lizards were captured in Qitai County; however, only one was detected as positive for *Leishmania* DNA.

**Table 3:** *Leishmania* DNA detection in lizards from different geographical districts

Districts	Number of lizards	Number of positive lizards	Prevalence	95% CI	Number of positive lizards (Infected by)							
					LS	LD	LT	LS + LD	LS + LT	LT + LD	LT + LS + LD	LTR + LS + LD
Jinghe County (S1)	26	23	88.46%	76.18–100.74%	23	9	1	9	1	–	–	1
Xinyuan County (S2)	8	4	50%	15.35–84.65%	3	1	0	–	–	–	–	–
Gongliu County (S3)	30	14	46.67%	28.82–64.52%	6	9	1	1	1	–	–	–
Nilke County (S4)	12	8	66.67%	40.00–93.34%	4	5	0	1	0	–	–	–
Tekes County (S5)	5	1	20%	−15.06–55.06%	0	0	1	–	–	–	–	–
Zhaosu County (S6)	1	0	0	0	0	0	0	–	–	–	–	–
Huocheng County (S7)	36	17	47.22%	30.91–63.53%	5	17	0	5	–	–	–	–
Fukang County (S8)	4	0	0	0	0	0	0	–	–	–	–	–
Qitai County (S9)	14	1	7.14%	−6.35–20.63%	0	1	0	–	–	–	–	–
Mori County(S10)	48	16	33.33%	19.99–46.67%	4	16	0	4	–	–	–	–
Barkol County (S11)	30	18	60%	42.47–77.53%	1	18	1	1	–	1	–	–
Yiwu County (S12)	59	45	76.27%	65.41–87.13%	27	36	11	19	9	5	4	–
Hami City (S13)	16	16	100%	100–100%	9	16	0	9	–	–	–	–
Dabancheng district (S14)	19	15	78.95%	60.62–97.28%	7	12	5	5	2	3	1	–
Akesai County (S15)	8	5	62.5%	28.95–96.05%	3	5	2	3	2	2	2	–
Total	316	183	57.91%	52.47–63.35%	92	145	22	57	15	11	7	1

*LS Leishmania sp.*, *LT Leishmania turanica*, *LD Leishmania donovani* complex, *LTR Leishmania tropica*

### Phylogenetic analysis

Sequencing of HSP70 and Cyt *b* was performed on *Leishmania* positive samples; the products were approximately 738 bp and 543 bp in length, respectively. Six clones had been sequenced for each positive sample to ensure that distinct sequences could be obtained for all infected *Leishmania*. All sequences were subjected for BLAST searching in GenBank and shown to be over 98% identity similar to *Leishmania*. In total, 195 HSP70 sequences and 299 Cyt *b* sequences were obtained and deposited in GenBank under accession numbers MH724314–MH724508 and MH724509–MH724807, respectively. Seventy-five HSP70-haplotypes (unique sequences) and 126 Cyt *b*-haplotypes were identified and used to conduct phylogenetic analyses with 22 and 31 reference sequences, respectively, downloaded from GenBank (see Additional files 1, 2 and 3).

**Fig. 1:**
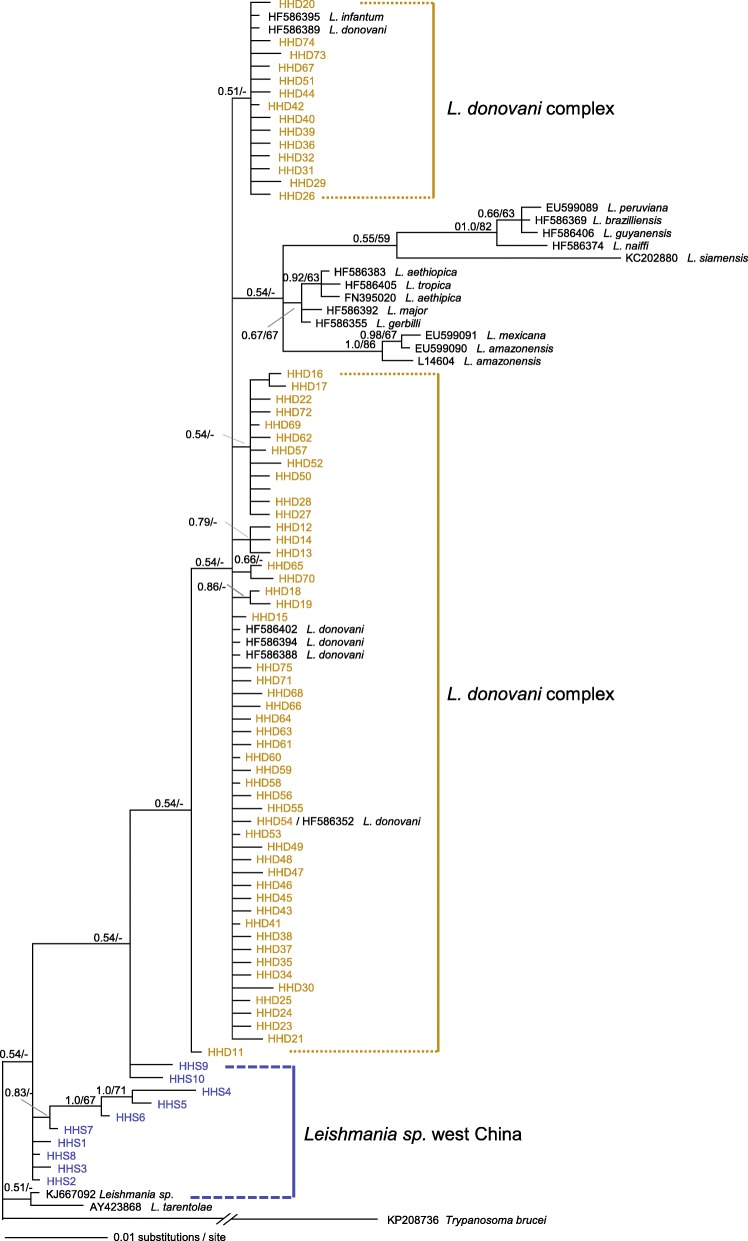
HSP70 gene majority-rule consensus tree inferred from Bayesian inference by using MrBayes v.3.2, associations with less than 0.5 posterior probability were collapsed. Bayesian posterior probabilities and maximum likelihood bootstrap values are shown. Dashes represent nodes with non-parametric bootstrap support lower than 50% or represent nodes not existed. Texts in colors are used to highlight haplotypes that were obtained in this study

**Fig. 2:**
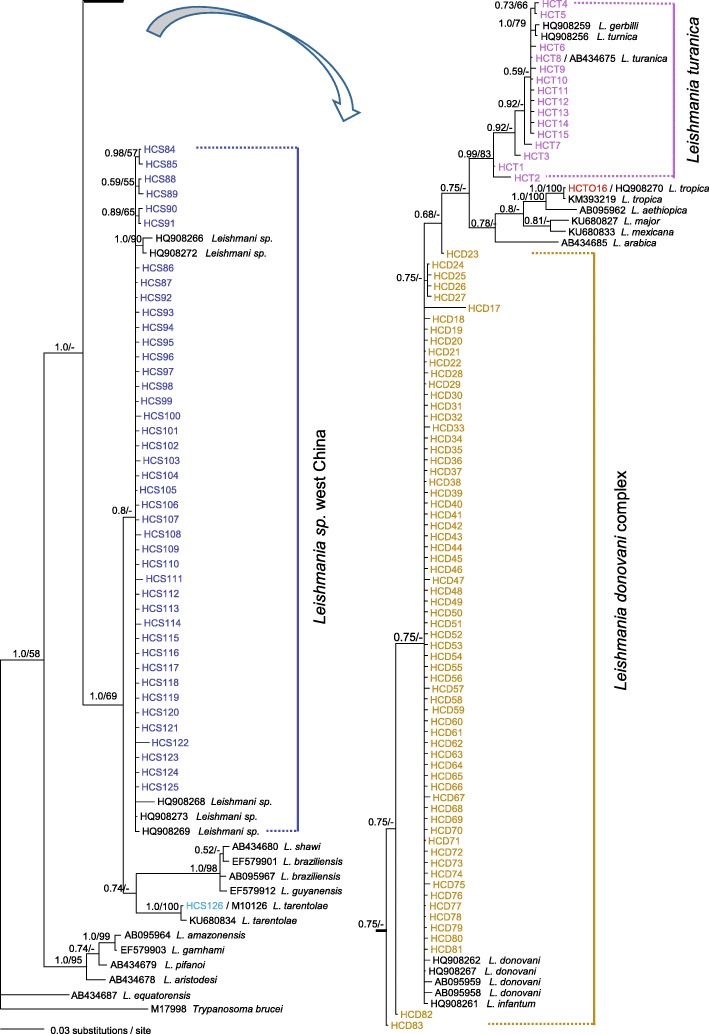
Cyt *b* gene majority-rule consensus tree inferred from Bayesian inference by using MrBayes v.3.2, associations with less than 0.5 posterior probability were collapsed. Bayesian posterior probabilities and maximum likelihood bootstrap values are shown. Dashes represent nodes with non-parametric bootstrap support lower than 50% or represent nodes not existed. Texts in colors are used to highlight haplotypes that were obtained in this study

Prior to the Bayesian phylogenetic analyses, the most adequate models of nucleotide substitution were selected by jModelTest: TrN + G for HSP70 and K80 + G for Cyt *b*, respectively. For both HSP70 and Cyt *b* dataset, Bayesian inference and ML analyses produced highly congruent topology, with only minor conflicts on very recent nodes. Thus, only the BI tree with both PP and BS from ML is presented (Figs. 1 and 2). As shown in Fig. 1, haplotypes HHS1–HHS10 were closely related to *Leishmania sp.* (accession number of KJ667092), and were referred to as *Leishmania sp.* west China in this study since most of them were found in Western China. Haplotypes HHD11–HHD75 were clustered with *L. donovani* and *L. infantum*, which were identified as *L. donovani* complex. Haplotype HHD42 shared the same sequence with reference sequence HF586393 (*L. infantum* isolate of MCAN/IL/97/LRC-L720). Similarly, haplotype HHD53 shared the same sequence with reference sequence HF586352 (*L. donovani* isolate of MHOM/MA/95/CRE72).

As shown in Fig. 2, haplotype HCTO16 shared the same sequence with reference HQ908257 and could be identified clearly as *L. tropica*. Haplotypes HCT1–HCT15 were clustered together with the reference strains of *L. turanica* and *L. gerbilli* with high support (PP = 0.99; BP = 83). Haplotypes HCD17–HCD83 were clustered with *L. donovani* and *L. infantum*, and were identified as *L. donovani* complex. Haplotypes HCS84–HSC125 were clustered with *Leishmania sp.* (PP = 0.8), being identified as *Leishmania sp*. west China. Haplotype HCT8 shared the same sequence with reference AB434675 (*L. turanica* MRHO/SU/80/CLONE3720); haplotype HCS126 shared the same sequence with M10126 (*L. tarentolae*).

**Fig. 3:**
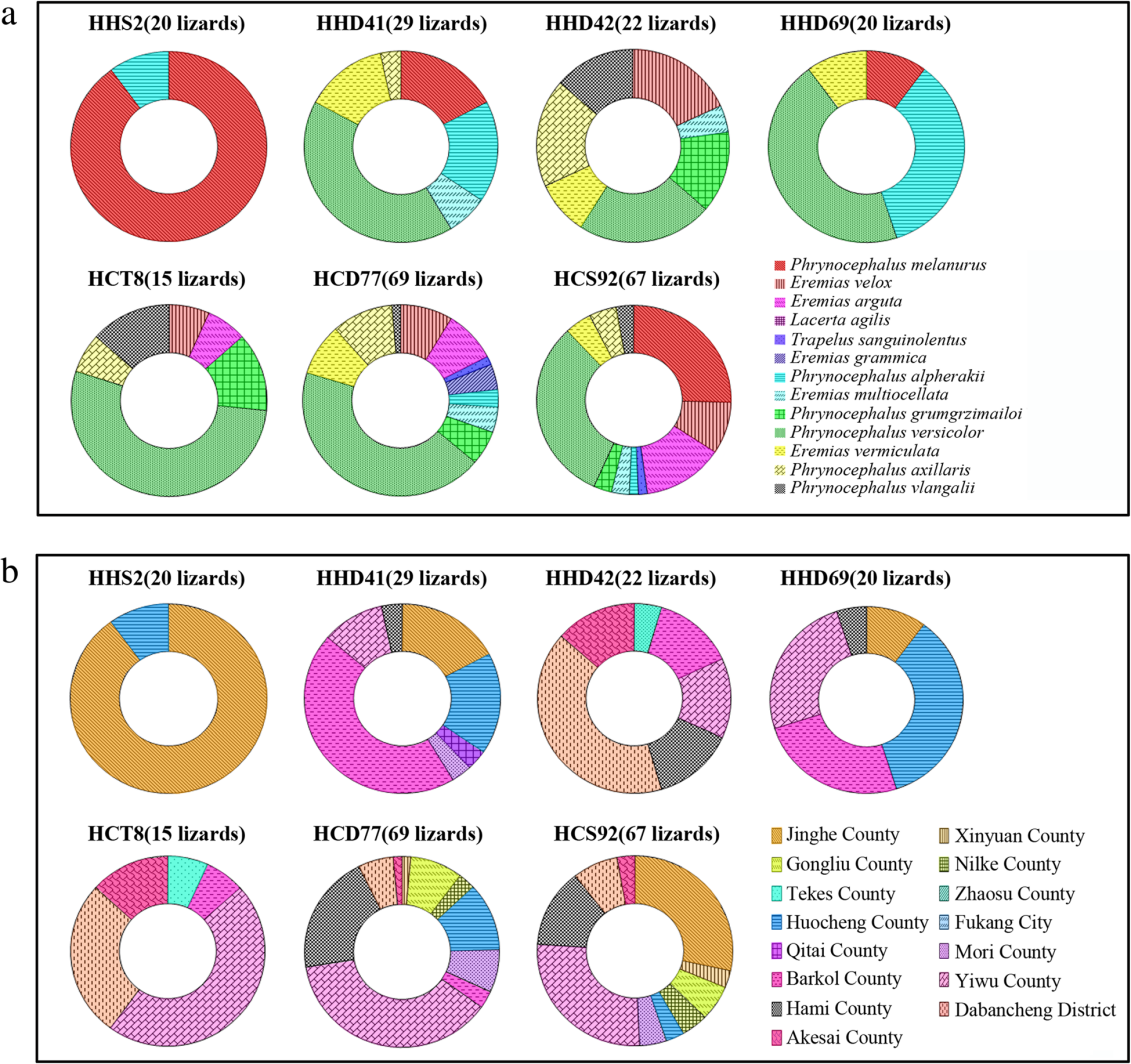
Common haplotypes obtained in this study. Different filled patterns represent the corresponding host affiliation (**a**) or geographical origin (**b**) from which the haplotype was sampled

Multiple haplotypes were found to be present in many lizards. As shown in Fig. 3, haplotypes HHS2, HHD41, HHD42 and HHD69 were present in 20, 29, 22 and 20 lizards from various districts and species, respectively. A total of 18 HSP70-haplotypes were present in at least two lizards (see Additional file 1). Similarly, haplotypes HCT8, HCD77 and HCS92 were present in 15, 69 and 67 lizards from various districts and species, respectively. A total of 19 Cyt *b*-haplotypes were present in at least two lizards (see Additional file 2).

A total of 47/183 PCR positive samples for *Leishmania* were amplified and sequenced successfully by both Cyt *b* and HSP70 markers (Additional files 1 and 2). In this study, *L. donovani* complex DNA was most frequently observed in lizards compared to other *Leishmania* species.

## Discussion

Leishmaniosis is still endemic in Northwest China. The reservoir for leishmaniosis, however, has puzzled researchers for over half a century. Several wild mammals in this area have been suspected, such as the wood mouse (*Apodemus sylvaticus*), midday gerbil (*Meriones meridianus*), and Yarkand hare (*Lepus yarkandensis*); however, attempts in detecting *Leishmania* parasites in these mammals have failed [20]. Positive *Leishmania* DNA was detected by PCR in blood samples from livestock (sheep, goat, cattle, donkey) in this area [20]. Yet the prevalence was less than 31% without more evidence accompanied with, such as the presence of the same parasite in reservoirs and humans, geographical distribution overlapped with vectors and so on [24], which is insufficient to support a reservoir role for these parasites.

The fact that reptiles can harbor *Leishmania* parasites is not unexpected to us. In the early 1970s, Belova noted that 21 species (including *Eremias velox*) from six lizard families may carry *Leishmania* promastigotes [7]. Subsequently, in 1982 Guan and his colleagues identified *Leishmania* amastigotes in lizard samples (*Teratoscincus przewalskii*) from Northwest China [25]. However, few *Leishmania* parasites from lizard have been virtually identified and categorized on species level. It is widely accepted that *Leishmania* parasites from reptiles are nonpathogenic to humans. Intriguingly, the role of reptiles in the epidemiology of *Leishmania* has yet to be determined. Various lizard species are known natural residents in Xinjiang, China, including lizards from Lacertidae (e.g. *E. velox* and *E. multiocellata*) and Agamidae (e.g. *P. axillaris*, *P. grumgrzimailoi*) [26]. Based on these facts, it is reasonable to assume that desert lizards in Northwest China may play a role in the epidemiology of leishmaniosis.

In our previous work, DNA of *L. donovani*, *L. tropica* and *Leishmania sp*. west China (the previous two species were known as human pathogen) were detected from lizards captured in Xinjiang [23]; the prevalence of *Leishmania* DNA was: *E. vermiculata* (22/34), *Eremias v. roborowskii* (14/25), *E. multiocellata* (6/6), *P. axillaris* (10/15), and *Tenuidactylus elongates* (1/1). In this study, 183 out of 316 lizards were detected to be positive for *Leishmania* DNA by PCR and various pathogenic *Leishmania* sequences were obtained. The frequency of zoonotic *Leishmania* spp. in lizard hosts is also presented. Nine additional species were involved in this study, and they appeared to be positive for *Leishmania* DNA. The prevalence of infection in different species of lizards is significant different (*P* < 0.05). Notably, the prevalence of *Leishmania* DNA was 90.91% (10/11) in *P. alpherakii*, much higher than in other reptiles (*P* < 0.05). However, it is not conclusively known that *P. alpherakii* is the preferential species harboring *Leishmania*, considering the fact that all specimens of *P. alpherakii* were captured from the same location. Compared to *E. velox* (47.06%), the prevalence of *Leishmania* DNA in *P. versicolor* (72.29%) is much higher (*P* < 0.05); this indicates that the difference of the prevalence may be related to host-specific preference of lizards, while both of them were collected from eight points.

Most regions in Xinjiang are arid deserts without sufficient water, along with various temperatures ranging from − 20 °C to 40 °C in summer. Small amounts of wild mammals here are observed in comparison to reptiles. Herpetological surveys revealed that arid-adapted lizards are widely distributed in Xinjiang and inhabited in ancient oases, deserts reclaimed by settlers for agricultural development and even around the counties [26]. No evidence has indicated the particular habitats present for the lizards yet. Although temperature is one of the environmental variables shaping the evolution and biology of reptiles, lizards could adjust their body temperature physiologically or through behavioral activity [27]. The preferred temperature and mean value may shift in different species of lizards [28]. This may be related to the difference in the ability of lizards to harbor parasites; no published data, however, support this speculation. Besides, ecology of the transmit vectors on the seasonal distributions and diets of the lizards should also be investigated to understand the specific role of different lizard hosts.

**Fig. 4:**
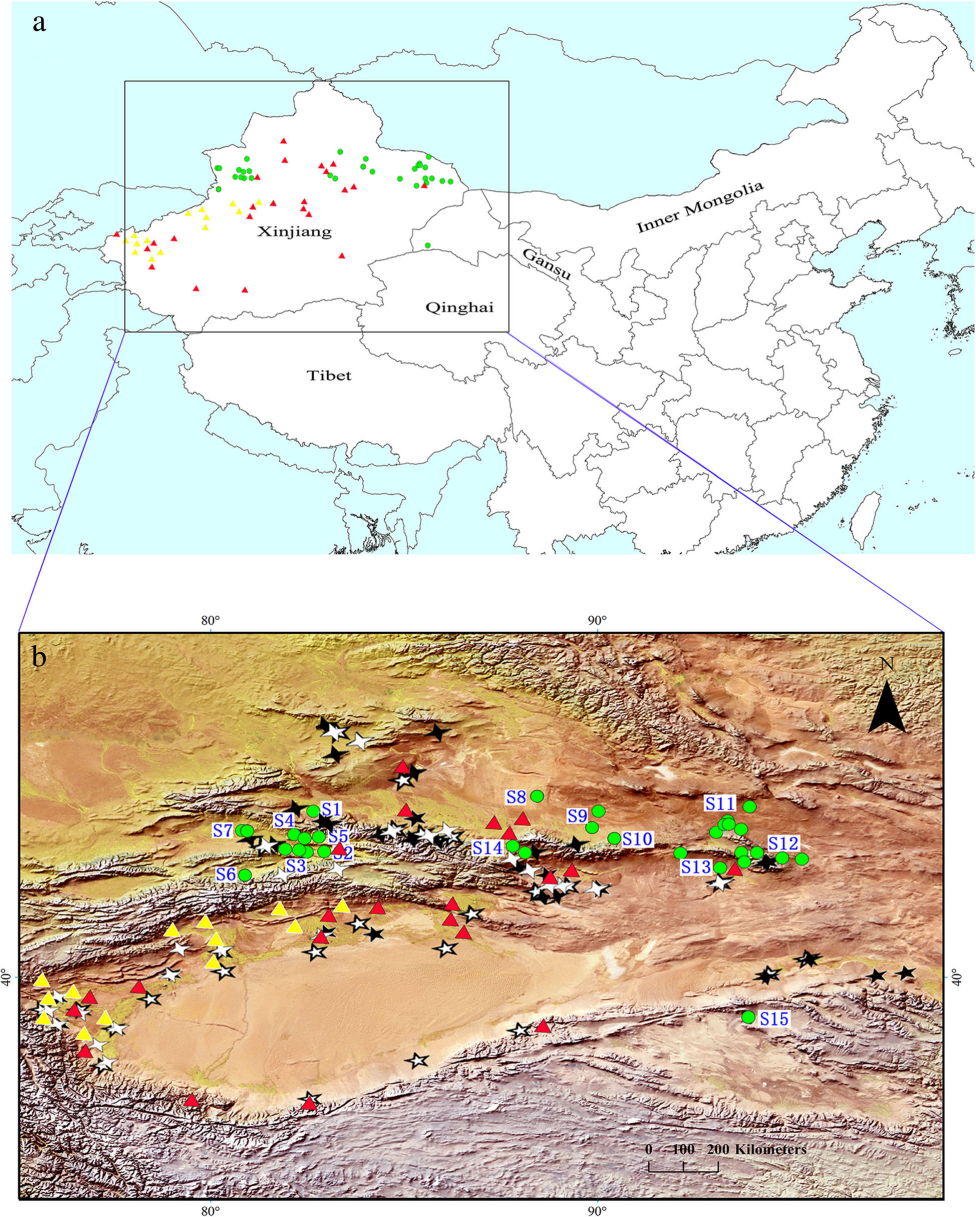
Sampling points of desert lizards in Northwest China, along with the current foci of endemicity of leishmaniosis. The above (**a**) shows the study area, sampling sites, and foci; the below (**b**) highlights the topography, sites numbers, and sandfly distribution pattern. Thirty one green solid circles () represent the points where lizards were captured in this study. The site numbers S1–S15 correspond to those in Table 1. Red solid triangles () represent desert-type zoonotic visceral leishmaniosis (DT-ZVL) endemic foci. Yellow solid triangles () represent anthroponotic type visceral leishmaniosis (AVL) endemic foci. Distribution of epidemic foci was collected and drawn according to the data published [15, 29–31]. Black and white stars represent the distributions of sandflies in Northwest China (★, *Phlebotomus mongolensis*; ☆, *Phlebotomus wui*; , *Phlebotomus alexandri*; , *Phlebotomus longiductus*), which were drawn according to the references [16, 32]. The image depicted in this figure is our own

All lizards in this study were captured from localities in or around the endemic foci of DZ-MCL [29–31], where the vector species *Phlebotomus longiductus*, *P. wui*, *P. alexandri*, and *P. mongolensis* are endemic (shown in Fig. 4) [16, 32]. In present study, DNA of four species of *Leishmania* was detected in the lizards, i.e. *L. turanica, L. tropica, L. donovani* complex and *Leishmania sp.* west China. While *Leishmania sp.*, belonging to *Sauroleishmania* (which is referred to as “lizard *Leishmania*”), allocated to the same cluster with *L. tarentolae*, is not beyond expectation. The other three species are known as the pathogenic *Leishmania*.

A total of 145 lizards (out of 183 *Leishmania* DNA positive lizards) were detected positive for *L. donovani* complex. It is widely known that *L. donovani* is the causative parasite for fatal visceral leishmaniosis (VL) in this region. From 2006 to 2012, 667 sporadic infantile VL cases, caused by *L. donovani* and *L. infantum,* were reported in Xinjiang [33]; several cases were in or close to our sampling sites.

*Leishmania turanica*, a well-known human pathogen transmitted by *P. mongolensis*, was also detected in this study. The species had been discovered in gerbils from Qitai (S8) and Fukang counties (S9) [34]. Human leishmaniosis, caused by this species with *P. mongolensis* as the transmitting vector, had also been reported in this area [34, 35]. It is assumed that there is a complete life cycle for *L. turanica* in this region with lizards serving as reservoir hosts.

*Leishmania tropica* is a protozoa causing anthroponotic cutaneous leishmaniosis as well as visceral leishmaniosis, and is transmitted by the vector of *Phlebotomus sergenti*. Only one lizard (*P. melanurus* from Jinhe County, with voucher number Guo4108, see Additional file 1) was detected to have positive DNA of *L. tropica* in present study. The positive detection rate for *L. tropica* was much lower than that in a previous study [23], which had the highest one (16/34) in *Eremias vermiculata* from the Turpan Depression. Fewer lizards detected to be positive for *L. tropica* may be related to the different foci where lizards were captured. Meanwhile, different PCR protocols should also be considered into consideration.

Mixed infections of *Leishmania* parasites are not uncommon in natural hosts from natural environments [36]. Consistent with a previous study [23], 77.6% (142/183) (95% CI, 71.55–83.64%) of lizards tested in this study were positive for at least two different haplotypes, and 37.7% (69/183) (95% CI, 30.68–44.73%) were positive for haplotypes of two *Leishmania* species, and 4.37% (8/183) (95% CI, 1.41–7.33%) were positive for haplotypes of three *Leishmania* species. The co-existence of different haplotypes or even species may indicate mixed infections. 18.41% (37/201) of the haplotypes were found in more than two lizards, and seven (HCT8, HCD77, HCS92, HHS2, HHD41, HHD42, HHD69) were found in more than fifteen lizards (Fig. 3). The shared haplotypes could be considered as common ancestry that is transmitted by vectors among lizards. These results also agree well with the notion of low host specificity of *Leishmania* in lizards [23], being similar to that observed in some other lizard parasites. For example, a recent study revealed that there is limited host specificity and no clear relation to the geographical distribution of *Hepatozoon* parasites in lizards from North Africa [37].

Interestingly, a large number of haplotypes, either based on HSP70 or Cyt *b* gene sequences, were clustered into a unique group. This group genetically diverged from any well-known pathogenic *Leishmania*s species. Instead, these haplotypes were clustered with nonpathogenic *L. tarentolae* in the phylogenetic tree inferred from Cyt *b* gene sequences, which is consistent with previous studies based on COII [38], Cyt *b* [39], 18S rRNA and 7SL RNA [40]. However, the DNA sequences analyzed in the previous studies were amplified from *Sergentomyia minuta* [41], canine leishmaniosis cases [42], and human leishmaniosis cases [43]. It is hypothesized that this undescribed *Leishmania* species may be a neglected pathogenic species or an optimum hybrid with genetic recombination of natural *Leishmania* spp. Unfortunately, the pathogenic role of this *Leishmania sp*. west China cannot be established until it is strictly confirmed according to Koch’s postulates. Meanwhile, further studies will endeavor to recover this *Leishmania sp*. west China from reptiles, which will be crucial for testing such a hypothesis.

Moreover, more evidences are needed to support the conclusion that lizards could serve as a potential reservoir for leishmaniosis, such as the presence of the same parasite in lizards, sandflies and humans, the maintains of parasites in skin lesions and blood at densities high enough to infect vectors and so on. Further studies on the attempt to isolate and culture parasites from the lizards and successful complete xenodiagnoses are also necessary.

## Conclusions

In this study we detected and identified *Leishmania* DNA in infected lizards. The results show a high prevalence of *Leishmania* in lizards including *Leishmania sp.* west China, *L. turanica*, *L. tarentolae*, *L. donovani* complex, and *L. tropica*. The pathogenic *Leishmania* species and commonly shared sequences in lizard species, from different locations, lend support to the potential reservoir role of lizards for human leishmaniosis. This study helps to understand the spread of epidemic leishmaniosis in Northwest China.

## Methods

### Ethics approval and consent to participate

All the procedures followed protocols approved by medical ethics committee of Sichuan University (No. K2018056) and were carried out under the National Guidelines for Experimental Animal Welfare (MOST of People’s Republic of China, 2006). We obtained permits to capture and use the lizards in this study from local legislation.

### Study area

In this study, lizards were captured alive by hand from July to August in 2015 from 31 sites in Northwest China where DT-ZVL is endemic (Fig. 4). This area is characteristic of arid deserts with an average altitude ranging from 400 to 2100 m above sea level. There are several known endemic foci of desert-type zoonotic visceral leishmaniosis (DT-ZVL) in or close to this area [15, 29–31]. All lizards were identified via morphological determination following the classification system of Zhao et al. [44]. The lizards were first anaesthetized with an intraperitoneal injection of 20 mg/ml of sodium pentobarbital. Then, blood samples were collected from the postorbital sinus directly in the freshly anaesthetized lizards using a heparinized glass capillary tube. After blood collection, all lizards were euthanized with an overdose of sodium pentobarbital delivered by intraperitoneal injection. Voucher specimens were fixed in 75% ethanol, and are deposited in the herpetological collection of Chengdu Institute of Biology, Chinese Academy of Sciences.

### DNA preparation, amplification, cloning and sequencing

 Total genomic DNA was extracted from blood samples using a commercial DNA extraction kit (TianGen Blood DNA Kit) according to the manufacturer’s protocol. Two sets of primers were used to detect *Leishmania* infections. The primers used to amplify Cyt *b* gene were L (5′-GTTACCATGTACAATGA TGTC-3′) and R (5′-AATTGTATATTATGATTTGTTTATTGTAG- 3′) [45]. The amplification of HSP70 gene was accomplished using primers L (5′-GACAACCGCCTCGTCAGGTTC-3′) and R (5′-GCAGATCGAGGTGACGTTCGAC-3′) [46]. The PCRs were conducted following previously published methods [45, 46]. Briefly, a total of 50 μL reaction volume was prepared, consisting of dNTPs, MgSO4, buffer and super fidelity enzyme, KOD (TOYOBO Bio-Technology Co., Ltd.); all components were mixed according to the manufacturer’s protocol. The PCR protocols for amplification were: 94 °C for 2 min followed by 40 cycles of 94 °C for 30 s, 55 °C for 30 s (Cyt *b*) or 57 °C for 30 s (HSP70) and 72 °C for 1 min, followed by a final extension step at 72 °C for 10 min. For each reaction, double distilled water was used as a negative control and DNA from *Leishmania* strain MHOM/CN/90/SC10H2 was used as a positive control. The purified PCR products were cloned into pGEM-T vector (Promega, USA) after purification. The ligated vectors were transformed into *Escherichia coli* DH5α competent cells, and the recombined plasmids were screened using the blue-white colony selection system. Suspected positive colonies were sequenced with the universal primers M13 on an ABI 3730 automated sequencer (Applied Biosystems, Inc.) at Tsingke Biological Technology Co., Ltd. (Chengdu, China).

### Phylogenetic analysis

Reference sequences were retrieved from GenBank. For HSP70 gene, 22 sequences from *Leishmania* strains were downloaded; 31 sequences were downloaded for Cyt *b* gene (see Additional file 3). Sequences from each gene were aligned using Clustal X v1.83 [47]. Distinct sequence types (haplotypes) were defined using DAMBE v5 [48].

Phylogenetic relationships among haplotypes were generated by Bayesian inference (BI) with MrBayes v3.2 [49]. Prior to BI analyses, we selected the optimal nucleotide substitution model using jModeltest v2.1.1 [50] under the Bayesian information criterion (BIC) for the dataset of HSP70 and Cyt *b*, respectively. Two independent runs were carried out with four Monte Carlo Markov chains (MCMCs) for 2 × 10^7^ generations with parameters and topologies sampled every 1000 generations. Convergence of the runs was assessed by the standard deviation of split frequencies (< 0.01). A 50% majority-rule consensus tree and posterior probability (PP) of clades were assessed by combining the sampled trees from the two independent runs after a 25% burn-in phase. *Trypanosoma brucei* was chosen as outgroup to root the tree, which was visualized using FigTree v1.4.3 [51]. To test the accuracy with which phylogeny could be inferred using the sequence data, the Bayesian approach was compared with the maximum likelihood (ML) method implemented using RAxML v8.2.4 [52] under the model of GTRGAMMA with 100 replicates with a complete random starting tree. For ML trees, node support was assessed by bootstrapping for 1000 replications.

### Statistical analyses

The relationships between the lizards’ geographical variables, host specific variable and the infecting *Leishmania* species were analyzed using the Excel (Office 2016) and IBM SPSS Statistic (version 21.0). The 95% confidence interval to proportion (CI) was calculated as: upper limit of confidence interval, P + 1.96*SQTR(p(1-p)/n); lower limit of confidence interval, P-1.96*SQTR(p(1-p)/n). A Chi square test performed to demonstrate the relationships between species of *Leishmania* and specific characteristics. *P* value < 0.05 was established to determine statistical significance.

## Supplementary information

**Additional file 1. **List of lizard samples, origin, detected *Leishmania* spp., and GenBank accession number for HSP70.

**Additional file 2. **List of lizard samples, origin, detected *Leishmania* spp., and GenBank accession numbers for Cyt *b*.

**Additional file 3.** List of other strains and sequences with accession numbers retrieved from GenBank.

## Data Availability

The sequences generated in the study are available in the GenBank repository under accession numbers MH724314–MH724807.

## Abbreviations

AVL: Anthroponotic type of visceral leishmaniosis
BI: Bayesian inference
BIC: Bayesian information criterion
BS: Bootstrap support
CI: Confidence interval
CL: Cutaneous leishmaniosis
Cyt *b*: Cytochrome *b*
DT-ZVL: Zoonotic desert type of visceral leishmaniosis
HSP70: heat shock protein 70
MCL: Muco-cutaneous leishmaniosis
MCMCs: Monte Carlo Markov chains
ML: Maximum likelihood
MT-ZVL: Zoonotic mountain type of visceral leishmaniosis
PCR: Polymerase chain reaction
PP: Posterior probability
VL: Visceral leishmaniosis
