# Supplementary material for: Pathogenic Leishmania spp. detected in lizards from Northwest China using molecular methods
Source: BMC Vet Res. 2019 Dec 9;15:446. doi: 10.1186/s12917-019-2174-4 (PMC6902407; doi:10.1186/s12917-019-2174-4)
Supplement: Supplementary file 1 — Additional file 1. List of lizard samples, origin, detected Leishmania spp., and GenBank accession number for HSP70. [file 12917_2019_2174_MOESM1_ESM.docx]

**Table S1** List of lizard samples, origin, detected *Leishmania* spp., and GenBank accession number for HSP70

| Haplotype number | Detected  *Leishmania* spp. | Voucher number | Lizard species | Origin | GenBank accession number | Sequence length (bp) |
| --- | --- | --- | --- | --- | --- | --- |
| HHS1 | *Leishmani sp.* | Guo4106 | *Phrynocephalus melanurus* | Point 1 | MH724314 | 738 |
| HHS2 | *Leishmani sp.* | Guo4100 | *Phrynocephalus melanurus* | Point 1 | MH724315 | 738 |
|  | *Leishmani sp.* | Guo4101 | *Phrynocephalus melanurus* | Point 1 | MH724316 | 738 |
|  | *Leishmani sp.* | Guo4103 | *Phrynocephalus melanurus* | Point 1 | MH724317 | 738 |
|  | *Leishmani sp.* | Guo4104 | *Phrynocephalus melanurus* | Point 1 | MH724318 | 738 |
|  | *Leishmani sp.* | Guo4105 | *Phrynocephalus melanurus* | Point 1 | MH724319 | 738 |
|  | *Leishmani sp.* | Guo4106 | *Phrynocephalus melanurus* | Point 1 | MH724320 | 738 |
|  | *Leishmani sp.* | Guo4107 | *Phrynocephalus melanurus* | Point 1 | MH724321 | 738 |
|  | *Leishmani sp.* | Guo4109 | *Phrynocephalus melanurus* | Point 1 | MH724322 | 738 |
|  | *Leishmani sp.* | Guo4111 | *Phrynocephalus melanurus* | Point 1 | MH724323 | 738 |
|  | *Leishmani sp.* | Guo4112 | *Phrynocephalus melanurus* | Point 1 | MH724324 | 738 |
|  | *Leishmani sp.* | Guo4113 | *Phrynocephalus melanurus* | Point 1 | MH724325 | 738 |
|  | *Leishmani sp.* | Guo4114 | *Phrynocephalus melanurus* | Point 1 | MH724326 | 738 |
|  | *Leishmani sp.* | Guo4115 | *Phrynocephalus melanurus* | Point 1 | MH724327 | 738 |
|  | *Leishmani sp.* | Guo4118 | *Phrynocephalus melanurus* | Point 1 | MH724328 | 738 |
|  | *Leishmani sp.* | Guo4119 | *Phrynocephalus melanurus* | Point 1 | MH724329 | 738 |
|  | *Leishmani sp.* | Guo4120 | *Phrynocephalus melanurus* | Point 1 | MH724330 | 738 |
|  | *Leishmani sp.* | Guo4122 | *Phrynocephalus melanurus* | Point 1 | MH724331 | 738 |
|  | *Leishmani sp.* | Guo4123 | *Phrynocephalus melanurus* | Point 1 | MH724332 | 738 |
|  | *Leishmani sp.* | Guo4236 | *Phrynocephalus alpherakii* | Point 11 | MH724333 | 738 |
|  | *Leishmani sp.* | Guo4238 | *Phrynocephalus alpherakii* | Point 11 | MH724334 | 738 |
| HHS3 | *Leishmani sp.* | Guo4122 | *Phrynocephalus melanurus* | Point 1 | MH724335 | 738 |
| HHS4 | *Leishmani sp.* | Guo4426 | *Phrynocephalus versicolor* | Point 18 | MH724336 | 738 |
| HHS5 | *Leishmani sp.* | Guo4341 | *Phrynocephalus grumgrzimailoi* | Point 15 | MH724337 | 738 |
|  | *Leishmani sp.* | Guo4635 | *Phrynocephalus versicolor* | Point 25 | MH724338 | 738 |
| HHS6 | *Leishmani sp.* | Guo4234 | *Phrynocephalus alpherakii* | Point 11 | MH724339 | 738 |
| HHS7 | *Leishmani sp.* | Guo4237 | *Phrynocephalus alpherakii* | Point 11 | MH724340 | 738 |
| HHS8 | *Leishmani sp.* | Guo4120 | *Phrynocephalus melanurus* | Point 1 | MH724341 | 738 |
|  | *Leishmani sp.* | Guo4232 | *Phrynocephalus alpherakii* | Point 11 | MH724342 | 738 |
|  | *Leishmani sp.* | Guo4114 | *Phrynocephalus melanurus* | Point 1 | MH724343 | 738 |
| HHS9 | *Leishmani sp.* | Guo4685 | *Phrynocephalus axillaris* | Point 29 | MH724344 | 738 |
| HHS10 | *Leishmani sp.* | Guo4122 | *Phrynocephalus melanurus* | Point 1 | MH724345 | 738 |
| HHD11 | *Leishmani donovani* | Guo4122 | *Phrynocephalus melanurus* | Point 1 | MH724346 | 738 |
| HHD12 | *Leishmani donovani* | Guo4426 | *Phrynocephalus versicolor* | Point 18 | MH724347 | 738 |
| HHD13 | *Leishmani donovani* | Guo4656 | *Phrynocephalus versicolor* | Point 25 | MH724348 | 738 |
| HHD14 | *Leishmani donovani* | Guo4632 | *Phrynocephalus versicolor* | Point 25 | MH724349 | 738 |
| HHD15 | *Leishmani donovani* | Guo4409 | *Phrynocephalus versicolor* | Point 17 | MH724350 | 735 |
| HHD16 | *Leishmani donovani* | Guo4233 | *Phrynocephalus alpherakii* | Point 11 | MH724351 | 738 |
|  | *Leishmani donovani* | Guo4237 | *Phrynocephalus alpherakii* | Point 11 | MH724352 | 738 |
| HHD17 | *Leishmani donovani* | Guo4237 | *Phrynocephalus alpherakii* | Point 11 | MH724353 | 738 |
| HHD18 | *Leishmani donovani* | Guo4381 | *Eremias multiocellata* | Point 15 | MH724354 | 738 |
|  | *Leishmani donovani* | Guo4411 | *Phrynocephalus versicolor* | Point 17 | MH724355 | 738 |
|  | *Leishmani donovani* | Guo4418 | *Phrynocephalus versicolor* | Point 18 | MH724356 | 738 |
|  | *Leishmani donovani* | Guo4426 | *Phrynocephalus versicolor* | Point 18 | MH724357 | 738 |
| HHD19 | *Leishmani donovani* | Guo4401 | *Eremias vermiculata* | Point 17 | MH724358 | 738 |
| HHD20 | *Leishmani donovani* | Guo4342 | *Phrynocephalus grumgrzimailoi* | Point 15 | MH724359 | 738 |
|  | *Leishmani donovani* | Guo4347 | *Phrynocephalus grumgrzimailoi* | Point 15 | MH724360 | 738 |
|  | *Leishmani donovani* | Guo4685 | *Phrynocephalus axillaris* | Point 29 | MH724361 | 738 |
| HHD21 | *Leishmani donovani* | Guo4401 | *Eremias vermiculata* | Point 17 | MH724362 | 738 |
| HHD22 | *Leishmani donovani* | Guo4439 | *Phrynocephalus versicolor* | Point 26 | MH724363 | 738 |
| HHD23 | *Leishmani donovani* | Guo4346 | *Phrynocephalus grumgrzimailoi* | Point 15 | MH724364 | 738 |
|  | *Leishmani donovani* | Guo4675 | *Eremias vermiculata* | Point 26 | MH724365 | 738 |
|  | *Leishmani donovani* | Guo4341 | *Phrynocephalus grumgrzimailoi* | Point 15 | MH724366 | 738 |
| HHD24 | *Leishmani donovani* | Guo4675 | *Eremias vermiculata* | Point 26 | MH724367 | 738 |
| HHD25 | *Leishmani donovani* | Guo4236 | *Phrynocephalus alpherakii* | Point 11 | MH724368 | 738 |
| HHD26 | *Leishmani donovani* | Guo4660 | *Phrynocephalus axillaris* | Point 28 | MH724369 | 738 |
| HHD27 | *Leishmani donovani* | Guo4384 | *Phrynocephalus versicolor* | Point 16 | MH724370 | 738 |
| HHD28 | *Leishmani donovani* | Guo4236 | *Phrynocephalus alpherakii* | Point 11 | MH724371 | 738 |
| HHD29 | *Leishmani donovani* | Guo4401 | *Eremias vermiculata* | Point 17 | MH724372 | 738 |
| HHD30 | *Leishmani donovani* | Guo4411 | *Phrynocephalus versicolor* | Point 17 | MH724373 | 738 |
| HHD31 | *Leishmani donovani* | Guo4656 | *Phrynocephalus versicolor* | Point 25 | MH724374 | 738 |
| HHD32 | *Leishmani donovani* | Guo4632 | *Phrynocephalus versicolor* | Point 25 | MH724375 | 738 |
| HHD33 | *Leishmani donovani* | Guo4387 | *Phrynocephalus versicolor* | Point 16 | MH724376 | 738 |
| HHD34 | *Leishmani donovani* | Guo4409 | *Phrynocephalus versicolor* | Point 17 | MH724377 | 738 |
| HHD35 | *Leishmani donovani* | Guo4102 | *Phrynocephalus melanurus* | Point 1 | MH724378 | 738 |
|  | *Leishmani donovani* | Guo4108 | *Phrynocephalus melanurus* | Point 1 | MH724379 | 738 |
|  | *Leishmani donovani* | Guo4233 | *Phrynocephalus alpherakii* | Point 11 | MH724380 | 738 |
|  | *Leishmani donovani* | Guo4387 | *Phrynocephalus versicolor* | Point 16 | MH724381 | 738 |
|  | *Leishmani donovani* | Guo4397 | *Phrynocephalus versicolor* | Point 16 | MH724382 | 738 |
|  | *Leishmani donovani* | Guo4425 | *Phrynocephalus versicolor* | Point 18 | MH724383 | 738 |
| HHD36 | *Leishmani donovani* | Guo4380 | *Eremias multiocellata* | Point 15 | MH724384 | 738 |
| HHD37 | *Leishmani donovani* | Guo4408 | *Eremias vermiculata* | Point 17 | MH724385 | 738 |
| HHD38 | *Leishmani donovani* | Guo4102 | *Phrynocephalus melanurus* | Point 1 | MH724386 | 738 |
| HHD39 | *Leishmani donovani* | Guo4675 | *Eremias vermiculata* | Point 26 | MH724387 | 738 |
| HHD40 | *Leishmani donovani* | Guo4706 | *Phrynocephalus vlangalii* | Point 31 | MH724388 | 738 |
| HHD41 | *Leishmani donovani* | Guo4102 | *Phrynocephalus melanurus* | Point 1 | MH724389 | 738 |
|  | *Leishmani donovani* | Guo4105 | *Phrynocephalus melanurus* | Point 1 | MH724390 | 738 |
|  | *Leishmani donovani* | Guo4111 | *Phrynocephalus melanurus* | Point 1 | MH724391 | 738 |
|  | *Leishmani donovani* | Guo4113 | *Phrynocephalus melanurus* | Point 1 | MH724392 | 738 |
|  | *Leishmani donovani* | Guo4114 | *Phrynocephalus melanurus* | Point 1 | MH724393 | 738 |
|  | *Leishmani donovani* | Guo4230 | *Phrynocephalus alpherakii* | Point 11 | MH724394 | 738 |
|  | *Leishmani donovani* | Guo4233 | *Phrynocephalus alpherakii* | Point 11 | MH724395 | 738 |
|  | *Leishmani donovani* | Guo4235 | *Phrynocephalus alpherakii* | Point 11 | MH724396 | 738 |
|  | *Leishmani donovani* | Guo4237 | *Phrynocephalus alpherakii* | Point 11 | MH724397 | 738 |
|  | *Leishmani donovani* | Guo4238 | *Phrynocephalus alpherakii* | Point 11 | MH724398 | 738 |
|  | *Leishmani donovani* | Guo4255 | *Eremias multiocellata* | Point 13 | MH724399 | 738 |
|  | *Leishmani donovani* | Guo4381 | *Eremias multiocellata* | Point 15 | MH724400 | 738 |
|  | *Leishmani donovani* | Guo4385 | *Phrynocephalus versicolor* | Point 16 | MH724401 | 738 |
|  | *Leishmani donovani* | Guo4398 | *Phrynocephalus versicolor* | Point 16 | MH724402 | 738 |
|  | *Leishmani donovani* | Guo4399 | *Phrynocephalus versicolor* | Point 16 | MH724403 | 738 |
|  | *Leishmani donovani* | Guo4400 | *Eremias vermiculata* | Point 17 | MH724404 | 738 |
|  | *Leishmani donovani* | Guo4401 | *Eremias vermiculata* | Point 17 | MH724405 | 738 |
|  | *Leishmani donovani* | Guo4408 | *Eremias vermiculata* | Point 17 | MH724406 | 738 |
|  | *Leishmani donovani* | Guo4409 | *Phrynocephalus versicolor* | Point 17 | MH724407 | 738 |
|  | *Leishmani donovani* | Guo4411 | *Phrynocephalus versicolor* | Point 17 | MH724408 | 738 |
|  | *Leishmani donovani* | Guo4419 | *Phrynocephalus versicolor* | Point 18 | MH724409 | 738 |
|  | *Leishmani donovani* | Guo4420 | *Phrynocephalus versicolor* | Point 18 | MH724410 | 738 |
|  | *Leishmani donovani* | Guo4421 | *Phrynocephalus versicolor* | Point 19 | MH724411 | 738 |
|  | *Leishmani donovani* | Guo4425 | *Phrynocephalus versicolor* | Point 18 | MH724412 | 738 |
|  | *Leishmani donovani* | Guo4426 | *Phrynocephalus versicolor* | Point 18 | MH724413 | 738 |
|  | *Leishmani donovani* | Guo4430 | *Phrynocephalus versicolor* | Point 21 | MH724414 | 738 |
|  | *Leishmani donovani* | Guo4610 | *Phrynocephalus versicolor* | Point 23 | MH724415 | 738 |
|  | *Leishmani donovani* | Guo4617 | *Eremias vermiculata* | Point 22 | MH724416 | 738 |
|  | *Leishmani donovani* | Guo4620 | *Phrynocephalus axillaris* | Point 27 | MH724417 | 738 |
| HHD42 | *Leishmani donovani* | Guo4381 | *Eremias multiocellata* | Point 15 | MH724418 | 738 |
|  | *Leishmani donovani* | Guo4401 | *Eremias vermiculata* | Point 17 | MH724419 | 738 |
|  | *Leishmani donovani* | Guo4409 | *Phrynocephalus versicolor* | Point 17 | MH724420 | 738 |
|  | *Leishmani donovani* | Guo4418 | *Phrynocephalus versicolor* | Point 18 | MH724421 | 738 |
|  | *Leishmani donovani* | Guo4601 | *Phrynocephalus versicolor* | Point 23 | MH724422 | 738 |
|  | *Leishmani donovani* | Guo4602 | *Phrynocephalus versicolor* | Point 23 | MH724423 | 738 |
|  | *Leishmani donovani* | Guo4656 | *Phrynocephalus versicolor* | Point 25 | MH724424 | 738 |
|  | *Leishmani donovani* | Guo4660 | *Phrynocephalus axillaris* | Point 28 | MH724425 | 738 |
|  | *Leishmani donovani* | Guo4665 | *Phrynocephalus axillaris* | Point 26 | MH724426 | 738 |
|  | *Leishmani donovani* | Guo4673 | *Eremias vermiculata* | Point 26 | MH724427 | 738 |
|  | *Leishmani donovani* | Guo4676 | *Eremias velox* | Point 29 | MH724428 | 738 |
|  | *Leishmani donovani* | Guo4677 | *Eremias velox* | Point 29 | MH724429 | 738 |
|  | *Leishmani donovani* | Guo4679 | *Eremias velox* | Point 29 | MH724430 | 738 |
|  | *Leishmani donovani* | Guo4684 | *Phrynocephalus axillaris* | Point 29 | MH724431 | 738 |
|  | *Leishmani donovani* | Guo4685 | *Phrynocephalus axillaris* | Point 29 | MH724432 | 738 |
|  | *Leishmani donovani* | Guo4688 | *Eremias velox* | Point 30 | MH724433 | 738 |
|  | *Leishmani donovani* | Guo4691 | *Phrynocephalus grumgrzimailoi* | Point 30 | MH724434 | 738 |
|  | *Leishmani donovani* | Guo4693 | *Phrynocephalus grumgrzimailoi* | Point 30 | MH724435 | 738 |
|  | *Leishmani donovani* | Guo4696 | *Phrynocephalus grumgrzimailoi* | Point 30 | MH724436 | 738 |
|  | *Leishmani donovani* | Guo4699 | *Phrynocephalus vlangalii* | Point 31 | MH724437 | 738 |
|  | *Leishmani donovani* | Guo4704 | *Phrynocephalus vlangalii* | Point 31 | MH724438 | 738 |
|  | *Leishmani donovani* | Guo4706 | *Phrynocephalus vlangalii* | Point 31 | MH724439 | 738 |
| HHD43 | *Leishmani donovani* | Guo4699 | *Phrynocephalus vlangalii* | Point 31 | MH724440 | 738 |
| HHD44 | *Leishmani donovani* | Guo4696 | *Phrynocephalus grumgrzimailoi* | Point 30 | MH724441 | 738 |
| HHD45 | *Leishmani donovani* | Guo4426 | *Phrynocephalus versicolor* | Point 18 | MH724442 | 738 |
| HHD46 | *Leishmani donovani* | Guo4399 | *Phrynocephalus versicolor* | Point 16 | MH724443 | 738 |
| HHD47 | *Leishmani donovani* | Guo4408 | *Eremias vermiculata* | Point 17 | MH724444 | 738 |
| HHD48 | *Leishmani donovani* | Guo4660 | *Phrynocephalus axillaris* | Point 28 | MH724445 | 738 |
| HHD49 | *Leishmani donovani* | Guo4408 | *Eremias vermiculata* | Point 17 | MH724446 | 738 |
| HHD50 | *Leishmani donovani* | Guo4387 | *Phrynocephalus versicolor* | Point 16 | MH724447 | 738 |
| HHD51 | *Leishmani donovani* | Guo4693 | *Phrynocephalus grumgrzimailoi* | Point 30 | MH724448 | 738 |
| HHD52 | *Leishmani donovani* | Guo4421 | *Phrynocephalus versicolor* | Point 19 | MH724449 | 738 |
| HHD53 | *Leishmani donovani* | Guo4381 | *Eremias multiocellata* | Point 15 | MH724450 | 738 |
|  | *Leishmani donovani* | Guo4408 | *Eremias vermiculata* | Point 17 | MH724451 | 738 |
|  | *Leishmani donovani* | Guo4409 | *Phrynocephalus versicolor* | Point 17 | MH724452 | 738 |
|  | *Leishmani donovani* | Guo4411 | *Phrynocephalus versicolor* | Point 17 | MH724453 | 738 |
|  | *Leishmani donovani* | Guo4418 | *Phrynocephalus versicolor* | Point 18 | MH724454 | 738 |
|  | *Leishmani donovani* | Guo4426 | *Phrynocephalus versicolor* | Point 18 | MH724455 | 738 |
|  | *Leishmani donovani* | Guo4601 | *Phrynocephalus versicolor* | Point 23 | MH724456 | 738 |
|  | *Leishmani donovani* | Guo4635 | *Phrynocephalus versicolor* | Point 25 | MH724457 | 738 |
|  | *Leishmani donovani* | Guo4679 | *Eremias velox* | Point 29 | MH724458 | 738 |
|  | *Leishmani donovani* | Guo4699 | *Phrynocephalus vlangalii* | Point 31 | MH724459 | 738 |
| HHD54 | *Leishmani donovani* | Guo4328 | *Eremias multiocellata* | Point 15 | MH724460 | 738 |
|  | *Leishmani donovani* | Guo4380 | *Eremias multiocellata* | Point 15 | MH724461 | 738 |
| HHD55 | *Leishmani donovani* | Guo4675 | *Eremias vermiculata* | Point 26 | MH724462 | 738 |
| HHD56 | *Leishmani donovani* | Guo4420 | *Phrynocephalus versicolor* | Point 18 | MH724463 | 738 |
| HHD57 | *Leishmani donovani* | Guo4230 | *Phrynocephalus alpherakii* | Point 11 | MH724464 | 738 |
|  | *Leishmani donovani* | Guo4235 | *Phrynocephalus alpherakii* | Point 11 | MH724465 | 738 |
|  | *Leishmani donovani* | Guo4236 | *Phrynocephalus alpherakii* | Point 11 | MH724466 | 738 |
|  | *Leishmani donovani* | Guo4238 | *Phrynocephalus alpherakii* | Point 11 | MH724467 | 738 |
| HHD58 | *Leishmani donovani* | Guo4409 | *Phrynocephalus versicolor* | Point 17 | MH724468 | 735 |
| HHD59 | *Leishmani donovani* | Guo4102 | *Phrynocephalus melanurus* | Point 1 | MH724469 | 738 |
| HHD60 | *Leishmani donovani* | Guo4109 | *Phrynocephalus melanurus* | Point 1 | MH724470 | 735 |
| HHD61 | *Leishmani donovani* | Guo4411 | *Phrynocephalus versicolor* | Point 17 | MH724471 | 738 |
| HHD62 | *Leishmani donovani* | Guo4232 | *Phrynocephalus alpherakii* | Point 11 | MH724472 | 738 |
| HHD63 | *Leishmani donovani* | Guo4411 | *Phrynocephalus versicolor* | Point 17 | MH724473 | 738 |
| HHD64 | *Leishmani donovani* | Guo4238 | *Phrynocephalus alpherakii* | Point 11 | MH724474 | 738 |
|  | *Leishmani donovani* | Guo4329 | *Eremias multiocellata* | Point 15 | MH724475 | 738 |
| HHD65 | *Leishmani donovani* | Guo4111 | *Phrynocephalus melanurus* | Point 1 | MH724476 | 738 |
|  | *Leishmani donovani* | Guo4405 | *Eremias vermiculata* | Point 17 | MH724477 | 738 |
| HHD66 | *Leishmani donovani* | Guo4119 | *Phrynocephalus melanurus* | Point 1 | MH724478 | 738 |
| HHD67 | *Leishmani donovani* | Guo4660 | *Phrynocephalus axillaris* | Point 28 | MH724479 | 738 |
| HHD68 | *Leishmani donovani* | Guo4420 | *Phrynocephalus versicolor* | Point 18 | MH724480 | 738 |
| HHD69 | *Leishmani donovani* | Guo4102 | *Phrynocephalus melanurus* | Point 1 | MH724481 | 738 |
|  | *Leishmani donovani* | Guo4114 | *Phrynocephalus melanurus* | Point 1 | MH724482 | 738 |
|  | *Leishmani donovani* | Guo4230 | *Phrynocephalus alpherakii* | Point 11 | MH724483 | 738 |
|  | *Leishmani donovani* | Guo4232 | *Phrynocephalus alpherakii* | Point 11 | MH724484 | 738 |
|  | *Leishmani donovani* | Guo4233 | *Phrynocephalus alpherakii* | Point 11 | MH724485 | 738 |
|  | *Leishmani donovani* | Guo4234 | *Phrynocephalus alpherakii* | Point 11 | MH724486 | 738 |
|  | *Leishmani donovani* | Guo4235 | *Phrynocephalus alpherakii* | Point 11 | MH724487 | 738 |
|  | *Leishmani donovani* | Guo4236 | *Phrynocephalus alpherakii* | Point 11 | MH724488 | 738 |
|  | *Leishmani donovani* | Guo4238 | *Phrynocephalus alpherakii* | Point 11 | MH724489 | 738 |
|  | *Leishmani donovani* | Guo4397 | *Phrynocephalus versicolor* | Point 16 | MH724490 | 738 |
|  | *Leishmani donovani* | Guo4399 | *Phrynocephalus versicolor* | Point 16 | MH724491 | 738 |
|  | *Leishmani donovani* | Guo4401 | *Eremias vermiculata* | Point 17 | MH724492 | 738 |
|  | *Leishmani donovani* | Guo4409 | *Phrynocephalus versicolor* | Point 17 | MH724493 | 738 |
|  | *Leishmani donovani* | Guo4419 | *Phrynocephalus versicolor* | Point 18 | MH724494 | 738 |
|  | *Leishmani donovani* | Guo4439 | *Phrynocephalus versicolor* | Point 26 | MH724495 | 738 |
|  | *Leishmani donovani* | Guo4599 | *Phrynocephalus versicolor* | Point 23 | MH724496 | 738 |
|  | *Leishmani donovani* | Guo4600 | *Phrynocephalus versicolor* | Point 23 | MH724497 | 738 |
|  | *Leishmani donovani* | Guo4610 | *Phrynocephalus versicolor* | Point 23 | MH724498 | 738 |
|  | *Leishmani donovani* | Guo4617 | *Eremias vermiculata* | Point 22 | MH724499 | 738 |
|  | *Leishmani donovani* | Guo4630 | *Phrynocephalus versicolor* | Point 25 | MH724500 | 738 |
| HHD70 | *Leishmani donovani* | Guo4119 | *Phrynocephalus melanurus* | Point 1 | MH724501 | 738 |
|  | *Leishmani donovani* | Guo4419 | *Phrynocephalus versicolor* | Point 18 | MH724502 | 738 |
| HHD71 | *Leishmani donovani* | Guo4620 | *Phrynocephalus axillaris* | Point 27 | MH724503 | 738 |
| HHD72 | *Leishmani donovani* | Guo4331 | *Eremias multiocellata* | Point 15 | MH724504 | 738 |
|  | *Leishmani donovani* | Guo4439 | *Phrynocephalus versicolor* | Point 26 | MH724505 | 738 |
| HHD73 | *Leishmani donovani* | Guo4408 | *Eremias vermiculata* | Point 17 | MH724506 | 738 |
| HHD74 | *Leishmani donovani* | Guo4685 | *Phrynocephalus axillaris* | Point 29 | MH724507 | 738 |
| HHD75 | *Leishmani donovani* | Guo4409 | *Phrynocephalus versicolor* | Point 17 | MH724508 | 738 |

Note: *Leishmani donovani* refers *to* the *L. donovani* complex.
