# Supplementary material for: Pathogenic Leishmania spp. detected in lizards from Northwest China using molecular methods
Source: BMC Vet Res. 2019 Dec 9;15:446. doi: 10.1186/s12917-019-2174-4 (PMC6902407; doi:10.1186/s12917-019-2174-4)
Supplement: Supplementary file 2 — Additional file 2. List of lizard samples, origin, detected Leishmania spp., and GenBank accession numbers for Cyt b. [file 12917_2019_2174_MOESM2_ESM.docx]

**Table S2** List of lizard samples, origin, isolated *Leishmania* spp., and GenBank accession numbers for Cyt *b*

| Haplotype number | Isolated  *Leishmania* spp. | Voucher number | Lizard species | Origin | GenBank accession | Sequence length (bp) |
| --- | --- | --- | --- | --- | --- | --- |
| HCT1 | *Leishmani turanica* | Guo4138 | *Eremias velox* | Point 3 | MH724509 | 543 |
| HCT2 | *Leishmani turanica* | Guo4138 | *Eremias velox* | Point 3 | MH724510 | 543 |
| HCT3 | *Leishmani turanica* | Guo4125 | *Eremias velox* | Point 1 | MH724511 | 543 |
| HCT4 | *Leishmani turanica* | Guo4616 | *Phrynocephalus versicolor* | Point 24 | MH724512 | 543 |
| HCT5 | *Leishmani turanica* | Guo4612 | *Phrynocephalus versicolor* | Point 23 | MH724513 | 543 |
| HCT6 | *Leishmani turanica* | Guo4676 | *Eremias velox* | Point 29 | MH724514 | 543 |
| HCT7 | *Leishmani turanica* | Guo4603 | *Phrynocephalus versicolor* | Point 23 | MH724515 | 543 |
| HCT8 | *Leishmani turanica* | Guo4172 | *Eremias arguta* | Point 8 | MH724516 | 543 |
|  | *Leishmani turanica* | Guo4409 | *Phrynocephalus versicolor* | Point 17 | MH724517 | 543 |
|  | *Leishmani turanica* | Guo4603 | *Phrynocephalus versicolor* | Point 23 | MH724518 | 543 |
|  | *Leishmani turanica* | Guo4604 | *Phrynocephalus versicolor* | Point 23 | MH724519 | 543 |
|  | *Leishmani turanica* | Guo4605 | *Phrynocephalus versicolor* | Point 23 | MH724520 | 543 |
|  | *Leishmani turanica* | Guo4609 | *Phrynocephalus versicolor* | Point 23 | MH724521 | 543 |
|  | *Leishmani turanica* | Guo4610 | *Phrynocephalus versicolor* | Point 23 | MH724522 | 543 |
|  | *Leishmani turanica* | Guo4612 | *Phrynocephalus versicolor* | Point 23 | MH724523 | 543 |
|  | *Leishmani turanica* | Guo4635 | *Phrynocephalus versicolor* | Point 25 | MH724524 | 543 |
|  | *Leishmani turanica* | Guo4676 | *Eremias velox* | Point 29 | MH724525 | 543 |
|  | *Leishmani turanica* | Guo4680 | *Phrynocephalus axillaris* | Point 29 | MH724526 | 543 |
|  | *Leishmani turanica* | Guo4695 | *Phrynocephalus grumgrzimailoi* | Point 30 | MH724527 | 543 |
|  | *Leishmani turanica* | Guo4696 | *Phrynocephalus grumgrzimailoi* | Point 30 | MH724528 | 543 |
|  | *Leishmani turanica* | Guo4704 | *Phrynocephalus vlangalii* | Point 31 | MH724529 | 543 |
|  | *Leishmani turanica* | Guo4706 | *Phrynocephalus vlangalii* | Point 31 | MH724530 | 543 |
| HCT9 | *Leishmani turanica* | Guo4696 | *Phrynocephalus grumgrzimailoi* | Point 30 | MH724531 | 543 |
| HCT10 | *Leishmani turanica* | Guo4609 | *Phrynocephalus versicolor* | Point 23 | MH724532 | 543 |
| HCT11 | *Leishmani turanica* | Guo4637 | *Phrynocephalus versicolor* | Point 25 | MH724533 | 543 |
| HCT12 | *Leishmani turanica* | Guo4696 | *Phrynocephalus grumgrzimailoi* | Point 30 | MH724534 | 543 |
| HCT13 | *Leishmani turanica* | Guo4597 | *Phrynocephalus versicolor* | Point 22 | MH724535 | 543 |
| HCT14 | *Leishmani turanica* | Guo4611 | *Phrynocephalus versicolor* | Point 23 | MH724536 | 543 |
| HCT15 | *Leishmani turanica* | Guo4679 | *Eremias velox* | Point 29 | MH724537 | 543 |
| HCTO16 | *Leishmani tropica* | Guo4108 | *Phrynocephalus melanurus* | Point 1 | MH724538 | 543 |
| HCD17 | *Leishmani donovani* | Guo4211 | *Eremias velox* | Point 11 | MH724539 | 543 |
| HCD18 | *Leishmani donovani* | Guo4614 | *Phrynocephalus versicolor* | Point 23 | MH724540 | 543 |
| HCD19 | *Leishmani donovani* | Guo4409 | *Phrynocephalus versicolor* | Point 17 | MH724541 | 543 |
| HCD20 | *Leishmani donovani* | Guo4661 | *Phrynocephalus versicolor* | Point 28 | MH724542 | 543 |
| HCD21 | *Leishmani donovani* | Guo4616 | *Phrynocephalus versicolor* | Point 24 | MH724543 | 543 |
|  | *Leishmani donovani* | Guo4635 | *Phrynocephalus versicolor* | Point 25 | MH724544 | 543 |
| HCD22 | *Leishmani donovani* | Guo4145 | *Eremias velox* | Point 5 | MH724545 | 543 |
|  | *Leishmani donovani* | Guo4409 | *Phrynocephalus versicolor* | Point 17 | MH724546 | 543 |
|  | *Leishmani donovani* | Guo4635 | *Phrynocephalus versicolor* | Point 25 | MH724547 | 543 |
| HCD23 | *Leishmani donovani* | Guo4211 | *Eremias velox* | Point 11 | MH724548 | 543 |
| HCD24 | *Leishmani donovani* | Guo4328 | *Eremias multiocellata* | Point 15 | MH724549 | 543 |
|  | *Leishmani donovani* | Guo4636 | *Phrynocephalus versicolor* | Point 25 | MH724550 | 543 |
| HCD25 | *Leishmani donovani* | Guo4186 | *Eremias arguta* | Point 4 | MH724551 | 543 |
| HCD26 | *Leishmani donovani* | Guo4192 | *Eremias arguta* | Point 4 | MH724552 | 543 |
| HCD27 | *Leishmani donovani* | Guo4630 | *Phrynocephalus versicolor* | Point 25 | MH724553 | 543 |
| HCD28 | *Leishmani donovani* | Guo4641 | *Phrynocephalus versicolor* | Point 21 | MH724554 | 543 |
| HCD29 | *Leishmani donovani* | Guo4145 | *Eremias velox* | Point 5 | MH724555 | 543 |
|  | *Leishmani donovani* | Guo4149 | *Eremias velox* | Point 5 | MH724556 | 543 |
| HCD30 | *Leishmani donovani* | Guo4656 | *Phrynocephalus versicolor* | Point 25 | MH724557 | 543 |
| HCD31 | *Leishmani donovani* | Guo4221 | *Eremias grammica* | Point 11 | MH724558 | 543 |
| HCD32 | *Leishmani donovani* | Guo4628 | *Phrynocephalus versicolor* | Point 25 | MH724559 | 543 |
|  | *Leishmani donovani* | Guo4658 | *Phrynocephalus versicolor* | Point 25 | MH724560 | 543 |
| HCD33 | *Leishmani donovani* | Guo4628 | *Phrynocephalus versicolor* | Point 25 | MH724561 | 543 |
| HCD34 | *Leishmani donovani* | Guo4660 | *Phrynocephalus versicolor* | Point 28 | MH724562 | 543 |
| HCD35 | *Leishmani donovani* | Guo4190 | *Eremias arguta* | Point 4 | MH724563 | 543 |
| HCD36 | *Leishmani donovani* | Guo4164 | *Eremias arguta* | Point 7 | MH724564 | 543 |
| HCD37 | *Leishmani donovani* | Guo4669 | *Phrynocephalus axillaris* | Point 26 | MH724565 | 543 |
| HCD38 | *Leishmani donovani* | Guo4231 | *Phrynocephalus alpherakii* | Point 11 | MH724566 | 543 |
|  | *Leishmani donovani* | Guo4234 | *Phrynocephalus alpherakii* | Point 11 | MH724567 | 543 |
|  | *Leishmani donovani* | Guo4340 | *Phrynocephalus grumgrzimailoi* | Point 15 | MH724568 | 543 |
|  | *Leishmani donovani* | Guo4344 | *Phrynocephalus grumgrzimailoi* | Point 15 | MH724569 | 543 |
| HCD39 | *Leishmani donovani* | Guo4170 | *Eremias arguta* | Point 7 | MH724570 | 543 |
| HCD40 | *Leishmani donovani* | Guo4619 | *Eremias vermiculata* | Point 22 | MH724571 | 543 |
| HCD41 | *Leishmani donovani* | Guo4646 | *Phrynocephalus versicolor* | Point 21 | MH724572 | 543 |
| HCD42 | *Leishmani donovani* | Guo4677 | *Eremias velox* | Point 29 | MH724573 | 543 |
| HCD43 | *Leishmani donovani* | Guo4673 | *Eremias vermiculata* | Point 26 | MH724574 | 543 |
| HCD44 | *Leishmani donovani* | Guo4639 | *Phrynocephalus versicolor* | Point 21 | MH724575 | 543 |
| HCD45 | *Leishmani donovani* | Guo4660 | *Phrynocephalus versicolor* | Point 28 | MH724576 | 543 |
| HCD46 | *Leishmani donovani* | Guo4137 | *Eremias velox* | Point 3 | MH724577 | 543 |
| HCD47 | *Leishmani donovani* | Guo4626 | *Phrynocephalus versicolor* | Point 25 | MH724578 | 543 |
| HCD48 | *Leishmani donovani* | Guo4640 | *Phrynocephalus versicolor* | Point 21 | MH724579 | 543 |
| HCD49 | *Leishmani donovani* | Guo4157 | *Eremias arguta* | Point 6 | MH724580 | 543 |
| HCD50 | *Leishmani donovani* | Guo4620 | *Phrynocephalus axillaris* | Point 27 | MH724581 | 543 |
| HCD51 | *Leishmani donovani* | Guo4165 | *Eremias arguta* | Point 7 | MH724582 | 543 |
| HCD52 | *Leishmani donovani* | Guo4687 | *Eremias velox* | Point 30 | MH724583 | 543 |
| HCD53 | *Leishmani donovani* | Guo4211 | *Eremias velox* | Point 11 | MH724584 | 543 |
|  | *Leishmani donovani* | Guo4216 | *Eremias grammica* | Point 11 | MH724585 | 543 |
| HCD54 | *Leishmani donovani* | Guo4158 | *Eremias arguta* | Point 7 | MH724586 | 543 |
|  | *Leishmani donovani* | Guo4228 | *Phrynocephalus alpherakii* | Point 11 | MH724587 | 543 |
| HCD55 | *Leishmani donovani* | Guo4378 | *Eremias multiocellata* | Point 15 | MH724588 | 543 |
| HCD56 | *Leishmani donovani* | Guo4629 | *Phrynocephalus versicolor* | Point 25 | MH724589 | 543 |
| HCD57 | *Leishmani donovani* | Guo4190 | *Eremias arguta* | Point 4 | MH724590 | 543 |
| HCD58 | *Leishmani donovani* | Guo4598 | *Phrynocephalus versicolor* | Point 23 | MH724591 | 543 |
| HCD59 | *Leishmani donovani* | Guo4659 | *Phrynocephalus versicolor* | Point 28 | MH724592 | 542 |
| HCD60 | *Leishmani donovani* | Guo4656 | *Phrynocephalus versicolor* | Point 25 | MH724593 | 543 |
| HCD61 | *Leishmani donovani* | Guo4640 | *Phrynocephalus versicolor* | Point 21 | MH724594 | 543 |
| HCD62 | *Leishmani donovani* | Guo4150 | *Eremias velox* | Point 5 | MH724595 | 543 |
|  | *Leishmani donovani* | Guo4216 | *Eremias grammica* | Point 11 | MH724596 | 543 |
| HCD63 | *Leishmani donovani* | Guo4624 | *Phrynocephalus versicolor* | Point 25 | MH724597 | 543 |
| HCD64 | *Leishmani donovani* | Guo4663 | *Phrynocephalus versicolor* | Point 28 | MH724598 | 543 |
| HCD65 | *Leishmani donovani* | Guo4700 | *Phrynocephalus vlangalii* | Point 31 | MH724599 | 543 |
| HCD66 | *Leishmani donovani* | Guo4637 | *Phrynocephalus versicolor* | Point 25 | MH724600 | 543 |
| HCD67 | *Leishmani donovani* | Guo4703 | *Phrynocephalus vlangalii* | Point 31 | MH724601 | 543 |
| HCD68 | *Leishmani donovani* | Guo4662 | *Phrynocephalus versicolor* | Point 28 | MH724602 | 543 |
| HCD69 | *Leishmani donovani* | Guo4619 | *Eremias vermiculata* | Point 22 | MH724603 | 543 |
| HCD70 | *Leishmani donovani* | Guo4192 | *Eremias arguta* | Point 4 | MH724604 | 543 |
|  | *Leishmani donovani* | Guo4670 | *Phrynocephalus axillaris* | Point 26 | MH724605 | 543 |
| HCD71 | *Leishmani donovani* | Guo4401 | *Eremias vermiculata* | Point 17 | MH724606 | 544 |
| HCD72 | *Leishmani donovani* | Guo4379 | *Eremias multiocellata* | Point 15 | MH724607 | 543 |
| HCD73 | *Leishmani donovani* | Guo4636 | *Phrynocephalus versicolor* | Point 25 | MH724608 | 543 |
| HCD74 | *Leishmani donovani* | Guo4677 | *Eremias velox* | Point 29 | MH724609 | 543 |
| HCD75 | *Leishmani donovani* | Guo4330 | *Eremias multiocellata* | Point 15 | MH724610 | 543 |
| HCD76 | *Leishmani donovani* | Guo4637 | *Phrynocephalus versicolor* | Point 25 | MH724611 | 543 |
| HCD77 | *Leishmani donovani* | Guo4126 | *Eremias arguta* | Point 2 | MH724612 | 543 |
|  | *Leishmani donovani* | Guo4145 | *Eremias velox* | Point 5 | MH724613 | 543 |
|  | *Leishmani donovani* | Guo4149 | *Eremias velox* | Point 5 | MH724614 | 543 |
|  | *Leishmani donovani* | Guo4150 | *Eremias velox* | Point 5 | MH724615 | 543 |
|  | *Leishmani donovani* | Guo4158 | *Eremias arguta* | Point 7 | MH724616 | 543 |
|  | *Leishmani donovani* | Guo4165 | *Eremias arguta* | Point 7 | MH724617 | 543 |
|  | *Leishmani donovani* | Guo4181 | *Eremias arguta* | Point 4 | MH724618 | 543 |
|  | *Leishmani donovani* | Guo4190 | *Eremias arguta* | Point 4 | MH724619 | 543 |
|  | *Leishmani donovani* | Guo4192 | *Eremias arguta* | Point 4 | MH724620 | 543 |
|  | *Leishmani donovani* | Guo4204 | *Eremias velox* | Point 10 | MH724621 | 543 |
|  | *Leishmani donovani* | Guo4211 | *Eremias velox* | Point 11 | MH724622 | 543 |
|  | *Leishmani donovani* | Guo4216 | *Eremias grammica* | Point 11 | MH724623 | 543 |
|  | *Leishmani donovani* | Guo4218 | *Eremias grammica* | Point 11 | MH724624 | 543 |
|  | *Leishmani donovani* | Guo4221 | *Eremias grammica* | Point 11 | MH724625 | 543 |
|  | *Leishmani donovani* | Guo4228 | *Phrynocephalus alpherakii* | Point 11 | MH724626 | 543 |
|  | *Leishmani donovani* | Guo4234 | *Phrynocephalus alpherakii* | Point 11 | MH724627 | 543 |
|  | *Leishmani donovani* | Guo4341 | *Phrynocephalus grumgrzimailoi* | Point 15 | MH724628 | 543 |
|  | *Leishmani donovani* | Guo4346 | *Phrynocephalus grumgrzimailoi* | Point 15 | MH724629 | 543 |
|  | *Leishmani donovani* | Guo4378 | *Eremias multiocellata* | Point 15 | MH724630 | 543 |
|  | *Leishmani donovani* | Guo4379 | *Eremias multiocellata* | Point 15 | MH724631 | 543 |
|  | *Leishmani donovani* | Guo4380 | *Eremias multiocellata* | Point 15 | MH724632 | 543 |
|  | *Leishmani donovani* | Guo4401 | *Eremias vermiculata* | Point 17 | MH724633 | 543 |
|  | *Leishmani donovani* | Guo4409 | *Phrynocephalus versicolor* | Point 17 | MH724634 | 543 |
|  | *Leishmani donovani* | Guo4598 | *Phrynocephalus versicolor* | Point 23 | MH724635 | 543 |
|  | *Leishmani donovani* | Guo4599 | *Phrynocephalus versicolor* | Point 23 | MH724636 | 543 |
|  | *Leishmani donovani* | Guo4600 | *Phrynocephalus versicolor* | Point 23 | MH724637 | 543 |
|  | *Leishmani donovani* | Guo4601 | *Phrynocephalus versicolor* | Point 23 | MH724638 | 543 |
|  | *Leishmani donovani* | Guo4606 | *Phrynocephalus versicolor* | Point 23 | MH724639 | 543 |
|  | *Leishmani donovani* | Guo4613 | *Phrynocephalus versicolor* | Point 23 | MH724640 | 543 |
|  | *Leishmani donovani* | Guo4618 | *Eremias vermiculata* | Point 22 | MH724641 | 543 |
|  | *Leishmani donovani* | Guo4619 | *Eremias vermiculata* | Point 22 | MH724642 | 543 |
|  | *Leishmani donovani* | Guo4620 | *Phrynocephalus axillaris* | Point 27 | MH724643 | 543 |
|  | *Leishmani donovani* | Guo4624 | *Phrynocephalus versicolor* | Point 25 | MH724644 | 543 |
|  | *Leishmani donovani* | Guo4625 | *Phrynocephalus versicolor* | Point 25 | MH724645 | 543 |
|  | *Leishmani donovani* | Guo4626 | *Phrynocephalus versicolor* | Point 25 | MH724646 | 543 |
|  | *Leishmani donovani* | Guo4627 | *Phrynocephalus versicolor* | Point 25 | MH724647 | 543 |
|  | *Leishmani donovani* | Guo4629 | *Phrynocephalus versicolor* | Point 25 | MH724648 | 543 |
|  | *Leishmani donovani* | Guo4630 | *Phrynocephalus versicolor* | Point 25 | MH724649 | 543 |
|  | *Leishmani donovani* | Guo4635 | *Phrynocephalus versicolor* | Point 25 | MH724650 | 543 |
|  | *Leishmani donovani* | Guo4637 | *Phrynocephalus versicolor* | Point 25 | MH724651 | 543 |
|  | *Leishmani donovani* | Guo4638 | *Phrynocephalus versicolor* | Point 21 | MH724652 | 543 |
|  | *Leishmani donovani* | Guo4639 | *Phrynocephalus versicolor* | Point 21 | MH724653 | 543 |
|  | *Leishmani donovani* | Guo4640 | *Phrynocephalus versicolor* | Point 21 | MH724654 | 543 |
|  | *Leishmani donovani* | Guo4641 | *Phrynocephalus versicolor* | Point 21 | MH724655 | 543 |
|  | *Leishmani donovani* | Guo4642 | *Phrynocephalus versicolor* | Point 21 | MH724656 | 543 |
|  | *Leishmani donovani* | Guo4646 | *Phrynocephalus versicolor* | Point 21 | MH724657 | 543 |
|  | *Leishmani donovani* | Guo4655 | *Phrynocephalus versicolor* | Point 25 | MH724658 | 543 |
|  | *Leishmani donovani* | Guo4656 | *Phrynocephalus versicolor* | Point 25 | MH724659 | 543 |
|  | *Leishmani donovani* | Guo4657 | *Phrynocephalus versicolor* | Point 25 | MH724660 | 543 |
|  | *Leishmani donovani* | Guo4658 | *Phrynocephalus versicolor* | Point 25 | MH724661 | 543 |
|  | *Leishmani donovani* | Guo4659 | *Phrynocephalus versicolor* | Point 28 | MH724662 | 543 |
|  | *Leishmani donovani* | Guo4660 | *Phrynocephalus versicolor* | Point 28 | MH724663 | 543 |
|  | *Leishmani donovani* | Guo4661 | *Phrynocephalus versicolor* | Point 28 | MH724664 | 543 |
|  | *Leishmani donovani* | Guo4662 | *Phrynocephalus versicolor* | Point 28 | MH724665 | 543 |
|  | *Leishmani donovani* | Guo4663 | *Phrynocephalus versicolor* | Point 28 | MH724666 | 543 |
|  | *Leishmani donovani* | Guo4664 | *Phrynocephalus versicolor* | Point 28 | MH724667 | 543 |
|  | *Leishmani donovani* | Guo4665 | *Phrynocephalus axillaris* | Point 26 | MH724668 | 543 |
|  | *Leishmani donovani* | Guo4666 | *Phrynocephalus versicolor* | Point 26 | MH724669 | 543 |
|  | *Leishmani donovani* | Guo4667 | *Phrynocephalus versicolor* | Point 26 | MH724670 | 543 |
|  | *Leishmani donovani* | Guo4669 | *Phrynocephalus axillaris* | Point 26 | MH724671 | 543 |
|  | *Leishmani donovani* | Guo4670 | *Phrynocephalus axillaris* | Point 26 | MH724672 | 543 |
|  | *Leishmani donovani* | Guo4674 | *Eremias vermiculata* | Point 26 | MH724673 | 543 |
|  | *Leishmani donovani* | Guo4675 | *Eremias vermiculata* | Point 26 | MH724674 | 543 |
|  | *Leishmani donovani* | Guo4677 | *Eremias velox* | Point 29 | MH724675 | 543 |
|  | *Leishmani donovani* | Guo4684 | *Phrynocephalus axillaris* | Point 29 | MH724676 | 543 |
|  | *Leishmani donovani* | Guo4690 | *Phrynocephalus grumgrzimailoi* | Point 30 | MH724677 | 543 |
|  | *Leishmani donovani* | Guo4697 | *Phrynocephalus grumgrzimailoi* | Point 30 | MH724678 | 543 |
|  | *Leishmani donovani* | Guo4698 | *Trapelus sanguinolentus* | Point 10 | MH724679 | 543 |
|  | *Leishmani donovani* | Guo4703 | *Phrynocephalus vlangalii* | Point 31 | MH724680 | 543 |
| HCD78 | *Leishmani donovani* | Guo4409 | *Phrynocephalus versicolor* | Point 17 | MH724681 | 542 |
| HCD79 | *Leishmani donovani* | Guo4327 | *Eremias multiocellata* | Point 15 | MH724682 | 543 |
| HCD80 | *Leishmani donovani* | Guo4600 | *Phrynocephalus versicolor* | Point 23 | MH724683 | 543 |
|  | *Leishmani donovani* | Guo4630 | *Phrynocephalus versicolor* | Point 25 | MH724684 | 543 |
| HCD81 | *Leishmani donovani* | Guo4184 | *Eremias arguta* | Point 4 | MH724685 | 543 |
|  | *Leishmani donovani* | Guo4227 | *Trapelus sanguinolentus* | Point 11 | MH724686 | 543 |
|  | *Leishmani donovani* | Guo4235 | *Phrynocephalus alpherakii* | Point 11 | MH724687 | 543 |
|  | *Leishmani donovani* | Guo4340 | *Phrynocephalus grumgrzimailoi* | Point 15 | MH724688 | 543 |
|  | *Leishmani donovani* | Guo4344 | *Phrynocephalus grumgrzimailoi* | Point 15 | MH724689 | 543 |
|  | *Leishmani donovani* | Guo4366 | *Phrynocephalus grumgrzimailoi* | Point 15 | MH724690 | 543 |
| HCD82 | *Leishmani donovani* | Guo4664 | *Phrynocephalus versicolor* | Point 28 | MH724691 | 543 |
| HCD83 | *Leishmani donovani* | Guo4186 | *Eremias arguta* | Point 4 | MH724692 | 543 |
| HCS84 | *Leishmani sp.* | Guo4684 | *Phrynocephalus axillaris* | Point 29 | MH724693 | 543 |
| HCS85 | *Leishmani sp.* | Guo4606 | *Phrynocephalus versicolor* | Point 23 | MH724694 | 543 |
| HCS86 | *Leishmani sp.* | Guo4127 | *Eremias arguta* | Point 2 | MH724695 | 543 |
| HCS87 | *Leishmani sp.* | Guo4148 | *Eremias velox* | Point 5 | MH724696 | 543 |
| HCS88 | *Leishmani sp.* | Guo4112 | *Phrynocephalus melanurus* | Point 1 | MH724697 | 543 |
|  | *Leishmani sp.* | Guo4114 | *Phrynocephalus melanurus* | Point 1 | MH724698 | 543 |
| HCS89 | *Leishmani sp.* | Guo4688 | *Eremias velox* | Point 30 | MH724699 | 543 |
| HCS90 | *Leishmani sp.* | Guo4689 | *Eremias velox* | Point 30 | MH724700 | 543 |
| HCS91 | *Leishmani sp.* | Guo4689 | *Eremias velox* | Point 30 | MH724701 | 543 |
| HCS92 | *Leishmani sp.* | Guo4100 | *Phrynocephalus melanurus* | Point 1 | MH724702 | 543 |
|  | *Leishmani sp.* | Guo4101 | *Phrynocephalus melanurus* | Point 1 | MH724703 | 543 |
|  | *Leishmani sp.* | Guo4102 | *Phrynocephalus melanurus* | Point 1 | MH724704 | 543 |
|  | *Leishmani sp.* | Guo4103 | *Phrynocephalus melanurus* | Point 1 | MH724705 | 543 |
|  | *Leishmani sp.* | Guo4104 | *Phrynocephalus melanurus* | Point 1 | MH724706 | 543 |
|  | *Leishmani sp.* | Guo4105 | *Phrynocephalus melanurus* | Point 1 | MH724707 | 543 |
|  | *Leishmani sp.* | Guo4107 | *Phrynocephalus melanurus* | Point 1 | MH724708 | 543 |
|  | *Leishmani sp.* | Guo4108 | *Phrynocephalus melanurus* | Point 1 | MH724709 | 543 |
|  | *Leishmani sp.* | Guo4111 | *Phrynocephalus melanurus* | Point 1 | MH724710 | 543 |
|  | *Leishmani sp.* | Guo4112 | *Phrynocephalus melanurus* | Point 1 | MH724711 | 543 |
|  | *Leishmani sp.* | Guo4113 | *Phrynocephalus melanurus* | Point 1 | MH724712 | 543 |
|  | *Leishmani sp.* | Guo4114 | *Phrynocephalus melanurus* | Point 1 | MH724713 | 543 |
|  | *Leishmani sp.* | Guo4118 | *Phrynocephalus melanurus* | Point 1 | MH724714 | 543 |
|  | *Leishmani sp.* | Guo4119 | *Phrynocephalus melanurus* | Point 1 | MH724715 | 543 |
|  | *Leishmani sp.* | Guo4120 | *Phrynocephalus melanurus* | Point 1 | MH724716 | 543 |
|  | *Leishmani sp.* | Guo4121 | *Phrynocephalus melanurus* | Point 1 | MH724717 | 543 |
|  | *Leishmani sp.* | Guo4122 | *Phrynocephalus melanurus* | Point 1 | MH724718 | 543 |
|  | *Leishmani sp.* | Guo4124 | *Eremias velox* | Point 1 | MH724719 | 543 |
|  | *Leishmani sp.* | Guo4125 | *Eremias velox* | Point 1 | MH724720 | 543 |
|  | *Leishmani sp.* | Guo4128 | *Eremias arguta* | Point 2 | MH724721 | 543 |
|  | *Leishmani sp.* | Guo4129 | *Eremias arguta* | Point 2 | MH724722 | 543 |
|  | *Leishmani sp.* | Guo4162 | *Eremias arguta* | Point 7 | MH724723 | 543 |
|  | *Leishmani sp.* | Guo4168 | *Eremias arguta* | Point 7 | MH724724 | 543 |
|  | *Leishmani sp.* | Guo4169 | *Eremias arguta* | Point 7 | MH724725 | 543 |
|  | *Leishmani sp.* | Guo4177 | *Eremias arguta* | Point 4 | MH724726 | 543 |
|  | *Leishmani sp.* | Guo4183 | *Eremias arguta* | Point 4 | MH724727 | 543 |
|  | *Leishmani sp.* | Guo4185 | *Eremias arguta* | Point 4 | MH724728 | 543 |
|  | *Leishmani sp.* | Guo4186 | *Eremias arguta* | Point 4 | MH724729 | 543 |
|  | *Leishmani sp.* | Guo4237 | *Phrynocephalus alpherakii* | Point 11 | MH724730 | 543 |
|  | *Leishmani sp.* | Guo4327 | *Eremias multiocellata* | Point 15 | MH724731 | 543 |
|  | *Leishmani sp.* | Guo4328 | *Eremias multiocellata* | Point 15 | MH724732 | 543 |
|  | *Leishmani sp.* | Guo4346 | *Phrynocephalus grumgrzimailoi* | Point 15 | MH724733 | 543 |
|  | *Leishmani sp.* | Guo4597 | *Phrynocephalus versicolor* | Point 22 | MH724734 | 543 |
|  | *Leishmani sp.* | Guo4602 | *Phrynocephalus versicolor* | Point 23 | MH724735 | 543 |
|  | *Leishmani sp.* | Guo4603 | *Phrynocephalus versicolor* | Point 23 | MH724736 | 543 |
|  | *Leishmani sp.* | Guo4607 | *Phrynocephalus versicolor* | Point 23 | MH724737 | 543 |
|  | *Leishmani sp.* | Guo4611 | *Phrynocephalus versicolor* | Point 23 | MH724738 | 543 |
|  | *Leishmani sp.* | Guo4612 | *Phrynocephalus versicolor* | Point 23 | MH724739 | 543 |
|  | *Leishmani sp.* | Guo4613 | *Phrynocephalus versicolor* | Point 23 | MH724740 | 543 |
|  | *Leishmani sp.* | Guo4615 | *Phrynocephalus versicolor* | Point 23 | MH724741 | 543 |
|  | *Leishmani sp.* | Guo4616 | *Phrynocephalus versicolor* | Point 24 | MH724742 | 543 |
|  | *Leishmani sp.* | Guo4618 | *Eremias vermiculata* | Point 22 | MH724743 | 543 |
|  | *Leishmani sp.* | Guo4625 | *Phrynocephalus versicolor* | Point 25 | MH724744 | 543 |
|  | *Leishmani sp.* | Guo4626 | *Phrynocephalus versicolor* | Point 25 | MH724745 | 543 |
|  | *Leishmani sp.* | Guo4636 | *Phrynocephalus versicolor* | Point 25 | MH724746 | 543 |
|  | *Leishmani sp.* | Guo4642 | *Phrynocephalus versicolor* | Point 21 | MH724747 | 543 |
|  | *Leishmani sp.* | Guo4655 | *Phrynocephalus versicolor* | Point 25 | MH724748 | 543 |
|  | *Leishmani sp.* | Guo4656 | *Phrynocephalus versicolor* | Point 25 | MH724749 | 543 |
|  | *Leishmani sp.* | Guo4657 | *Phrynocephalus versicolor* | Point 25 | MH724750 | 543 |
|  | *Leishmani sp.* | Guo4658 | *Phrynocephalus versicolor* | Point 25 | MH724751 | 543 |
|  | *Leishmani sp.* | Guo4659 | *Phrynocephalus versicolor* | Point 28 | MH724752 | 543 |
|  | *Leishmani sp.* | Guo4661 | *Phrynocephalus versicolor* | Point 28 | MH724753 | 543 |
|  | *Leishmani sp.* | Guo4662 | *Phrynocephalus versicolor* | Point 28 | MH724754 | 543 |
|  | *Leishmani sp.* | Guo4663 | *Phrynocephalus versicolor* | Point 28 | MH724755 | 543 |
|  | *Leishmani sp.* | Guo4664 | *Phrynocephalus versicolor* | Point 28 | MH724756 | 543 |
|  | *Leishmani sp.* | Guo4666 | *Phrynocephalus versicolor* | Point 26 | MH724757 | 543 |
|  | *Leishmani sp.* | Guo4670 | *Phrynocephalus axillaris* | Point 26 | MH724758 | 543 |
|  | *Leishmani sp.* | Guo4673 | *Eremias vermiculata* | Point 26 | MH724759 | 543 |
|  | *Leishmani sp.* | Guo4674 | *Eremias vermiculata* | Point 26 | MH724760 | 543 |
|  | *Leishmani sp.* | Guo4679 | *Eremias velox* | Point 29 | MH724761 | 543 |
|  | *Leishmani sp.* | Guo4687 | *Eremias velox* | Point 30 | MH724762 | 543 |
|  | *Leishmani sp.* | Guo4688 | *Eremias velox* | Point 30 | MH724763 | 543 |
|  | *Leishmani sp.* | Guo4689 | *Eremias velox* | Point 30 | MH724764 | 543 |
|  | *Leishmani sp.* | Guo4695 | *Phrynocephalus grumgrzimailoi* | Point 30 | MH724765 | 543 |
|  | *Leishmani sp.* | Guo4698 | *Trapelus sanguinolentus* | Point 10 | MH724766 | 543 |
|  | *Leishmani sp.* | Guo4700 | *Phrynocephalus vlangalii* | Point 31 | MH724767 | 543 |
|  | *Leishmani sp.* | Guo4706 | *Phrynocephalus vlangalii* | Point 31 | MH724768 | 543 |
| HCS93 | *Leishmani sp.* | Guo4595 | *Phrynocephalus versicolor* | Point 22 | MH724769 | 543 |
| HCS94 | *Leishmani sp.* | Guo4604 | *Phrynocephalus versicolor* | Point 23 | MH724770 | 543 |
| HCS95 | *Leishmani sp.* | Guo4128 | *Eremias arguta* | Point 2 | MH724771 | 543 |
| HCS96 | *Leishmani sp.* | Guo4595 | *Phrynocephalus versicolor* | Point 22 | MH724772 | 543 |
| HCS97 | *Leishmani sp.* | Guo4177 | *Eremias arguta* | Point 4 | MH724773 | 543 |
| HCS98 | *Leishmani sp.* | Guo4615 | *Phrynocephalus versicolor* | Point 23 | MH724774 | 543 |
| HCS99 | *Leishmani sp.* | Guo4673 | *Eremias vermiculata* | Point 26 | MH724775 | 543 |
| HCS100 | *Leishmani sp.* | Guo4704 | *Phrynocephalus vlangalii* | Point 31 | MH724776 | 543 |
| HCS101 | *Leishmani sp.* | Guo4611 | *Phrynocephalus versicolor* | Point 23 | MH724777 | 543 |
| HCS102 | *Leishmani sp.* | Guo4695 | *Phrynocephalus grumgrzimailoi* | Point 30 | MH724778 | 543 |
| HCS103 | *Leishmani sp.* | Guo4613 | *Phrynocephalus versicolor* | Point 23 | MH724779 | 543 |
| HCS104 | *Leishmani sp.* | Guo4602 | *Phrynocephalus versicolor* | Point 23 | MH724780 | 543 |
| HCS105 | *Leishmani sp.* | Guo4103 | *Phrynocephalus melanurus* | Point 1 | MH724781 | 538 |
| HCS106 | *Leishmani sp.* | Guo4666 | *Phrynocephalus versicolor* | Point 26 | MH724782 | 543 |
| HCS107 | *Leishmani sp.* | Guo4658 | *Phrynocephalus versicolor* | Point 25 | MH724783 | 543 |
| HCS108 | *Leishmani sp.* | Guo4655 | *Phrynocephalus versicolor* | Point 25 | MH724784 | 543 |
| HCS109 | *Leishmani sp.* | Guo4100 | *Phrynocephalus melanurus* | Point 1 | MH724785 | 543 |
| HCS110 | *Leishmani sp.* | Guo4607 | *Phrynocephalus versicolor* | Point 23 | MH724786 | 543 |
| HCS111 | *Leishmani sp.* | Guo4612 | *Phrynocephalus versicolor* | Point 23 | MH724787 | 542 |
| HCS112 | *Leishmani sp.* | Guo4170 | *Eremias arguta* | Point 7 | MH724788 | 543 |
| HCS113 | *Leishmani sp.* | Guo4632 | *Phrynocephalus versicolor* | Point 25 | MH724789 | 543 |
| HCS114 | *Leishmani sp.* | Guo4125 | *Eremias velox* | Point 1 | MH724790 | 543 |
| HCS115 | *Leishmani sp.* | Guo4177 | *Eremias arguta* | Point 4 | MH724791 | 543 |
|  | *Leishmani sp.* | Guo4603 | *Phrynocephalus versicolor* | Point 23 | MH724792 | 543 |
| HCS116 | *Leishmani sp.* | Guo4679 | *Eremias velox* | Point 29 | MH724793 | 543 |
| HCS117 | *Leishmani sp.* | Guo4626 | *Phrynocephalus versicolor* | Point 25 | MH724794 | 543 |
| HCS118 | *Leishmani sp.* | Guo4138 | *Eremias velox* | Point 3 | MH724795 | 543 |
|  | *Leishmani sp.* | Guo4169 | *Eremias arguta* | Point 7 | MH724796 | 543 |
|  | *Leishmani sp.* | Guo4610 | *Phrynocephalus versicolor* | Point 23 | MH724797 | 543 |
|  | *Leishmani sp.* | Guo4684 | *Phrynocephalus axillaris* | Point 29 | MH724798 | 543 |
| HCS119 | *Leishmani sp.* | Guo4657 | *Phrynocephalus versicolor* | Point 25 | MH724799 | 543 |
| HCS120 | *Leishmani sp.* | Guo4604 | *Phrynocephalus versicolor* | Point 23 | MH724800 | 543 |
| HCS121 | *Leishmani sp.* | Guo4641 | *Phrynocephalus versicolor* | Point 21 | MH724801 | 543 |
| HCS122 | *Leishmani sp.* | Guo4128 | *Eremias arguta* | Point 2 | MH724802 | 543 |
| HCS123 | *Leishmani sp.* | Guo4603 | *Phrynocephalus versicolor* | Point 23 | MH724803 | 543 |
| HCS124 | *Leishmani sp.* | Guo4613 | *Phrynocephalus versicolor* | Point 23 | MH724804 | 543 |
| HCS125 | *Leishmani sp.* | Guo4101 | *Phrynocephalus melanurus* | Point 1 | MH724805 | 543 |
| HCS126 | *Leishmani tarentolae* | Guo4601 | *Phrynocephalus versicolor* | Point 23 | MH724806 | 543 |
| HCS126 | *Leishmani tarentolae* | Guo4609 | *Phrynocephalus versicolor* | Point 23 | MH724807 | 543 |

Note: *Leishmani donovani* refers to the *L. donovani* complex.
