# Supplementary material for: Pathogenic Leishmania spp. detected in lizards from Northwest China using molecular methods
Source: BMC Vet Res. 2019 Dec 9;15:446. doi: 10.1186/s12917-019-2174-4 (PMC6902407; doi:10.1186/s12917-019-2174-4)
Supplement: Supplementary file 3 — Additional file 3. List of other strains and sequences with accession numbers retrieved from GenBank. [file 12917_2019_2174_MOESM3_ESM.docx]

**Table S3** List of other strains and sequences with accession numbers retrieved from GenBank

| Gene | Species | Isolate (WHO code) | GenBank accession number | Sequence length (bp) | References |
| --- | --- | --- | --- | --- | --- |
| cyt *b* | *Leishmania turanica* | MRHO/CN/88/KXG-2 | HQ908256 | 543 | R1 |
|  | *Leishmania gerbilli* | MGER/CN/60/GS-GER20 | HQ908259 | 543 | R1 |
|  | *Leishmania turanica* | MRHO/SU/80/CLONE3720 | AB434675 | 543 | R2 |
|  | *Leishmania tropica* | MHOM/SU/74/K27 | HQ908270 | 543 | R1 |
|  | *Leishmania tropica* | / | KM393219 | 546 | R3 |
|  | *Leishmania aethiopica* | MHOM/ET/72/L100 | AB095962 | 543 | R4 |
|  | *Leishmania major* | MHOM/SU/73/5ASKH | KU680827 | 543 | R5 |
|  | *Leishmania mexicana* | MNYC/BZ/62/M379 | KU680833 | 543 | R5 |
|  | *Leishmania arabica* | MPSA/SA/83/JISH220 | AB434685 | 543 | R2 |
|  | *Leishmania donovani* | IPHL/CN/77/XJ771 | HQ908267 | 543 | R1 |
|  | *Leishmania donovani* | MCAN/CN/60/GS1 | HQ908262 | 543 | R1 |
|  | *Leishmania donovani* | MHOM/BR/74/PP75 | AB095959 | 543 | R4 |
|  | *Leishmania infantum* | MHOM/CN/93/GS7 | HQ908261 | 543 | R1 |
|  | *Leishmania donovani* | MHOM/TN/80/IPT1 | AB095958 | 543 | R4 |
|  | *Leishmania sp.* | MHOM/CN/90/SC11 | HQ908272 | 543 | R1 |
|  | *Leishmania sp.* | MHOM/CN/80/XJ801 | HQ908266 | 543 | R1 |
|  | *Leishmania sp.* | MHOM/CN/90/SC10H2 | HQ908260 | 543 | R1 |
|  | *Leishmania sp.* | MCAN/CN/86/SC9 | HQ908268 | 543 | R1 |
|  | *Leishmania sp.* | MHOM/CN/83/GS2 | HQ908273 | 545 | R1 |
|  | *Leishmania sp.* | MHOM/CN/89/GS5 | HQ908269 | 546 | R1 |
|  | *Leishmania shawi* | MHOM/BR/79/M15065 | AB434680 | 543 | R2 |
|  | *Leishmania braziliensis* | MHOM/BR/84/LTB300 | EF579910 | 543 | R6 |
|  | *Leishmania braziliensis* | MHOM/EC/88/INH-03 | AB095967 | 543 | R4 |
|  | *Leishmania guyanensis* | MHOM/BR/75/M4147 | EF579912 | 543 | R6 |
|  | *Leishmania tarentolae* | / | KU680834 | 543 | R5 |
|  | *Leishmania tarentolae* | / | M10126 | 543 | R7 |
|  | *Leishmania amazonensis* | MHOM/BR/73/M2269 | AB095964 | 543 | R4 |
|  | *Leishmania garnhami* | MHOM/VE/76/JAP78 | EF579903 | 543 | R6 |
|  | *Leishmania pifanoi* | MHOM/VE/57/LL1 | AB434679 | 543 | R2 |
|  | *Leishmania equatorensis* | MSCI/EC/82/LSP-2 | AB434687 | 549 | R2 |
|  | *Trypanosoma brucei* | / | M17998 | 543 | R13 |
| Hsp70 | *Leishmania tarentolae* | / | AY423868 | 738 | R7 |
|  | *Leishmania sp.* | MHOM/GS/89/GS6 | KJ667092 | 738 | Unpublished |
|  | *Leishmania donovani* | MHOM/KE/89/EB59 | HF586402 | 738 | R9 |
|  | *Leishmania infantum* | MHOM/ES/2000/LLM938 | HF586395 | 738 | R9 |
|  | *Leishmania donovani* | MHOM/CN/00/WANGJIE-1 | HF586394 | 738 | R9 |
|  | *Leishmania infantum* | MCAN/IL/97/LRC-L720 | HF586393 | 738 | R9 |
|  | *Leishmania donovani* | MHOM/MA/95/CRE72 | HF586352 | 738 | R9 |
|  | *Leishmania donovani* | MHOM/SU/84/MARZ-KRIM | HF586389 | 738 | R9 |
|  | *Leishmania donovani* | IMRT/ET/90/TESHOME210 | HF586388 | 738 | R9 |
|  | *Leishmania aethiopica* | MHOM/ET/70/L96 | HF586383 | 738 | R9 |
|  | *Leishmania tropica* | MHOM/EG/90/LPN65 | HF586405 | 738 | R9 |
|  | *Leishmania aethiopica* | MHOM/ET/83/169-83 | FN395020 | 738 | R10 |
|  | *Leishmania major* | MHOM/JO/90/JH39 | HF586392 | 738 | R9 |
|  | *Leishmania gerbilli* | MRHO/SU/87/E-11 | HF586355 | 738 | R9 |
|  | *Leishmania amazonensis* | MHOM/BR/73/M2269 | EU599090 | 738 | R10 |
|  | *Leishmania mexicana* | MNYC/BZ/62/M379 | EU599091 | 738 | R10 |
|  | *Leishmania amazonensis* | MHOM/BR/77/LTB0016/C1S1 | L14601 | 738 | R11 |
|  | *Leishmania peruviana* | MHOM/PE/90/LCA08CL2 | EU599089 | 738 | R11 |
|  | *Leishmania guyanensis* | MHOM/GF/2004/GAE1 | HF586406 | 738 | R9 |
|  | *Leishmania braziliensis* | IWHI/BR/86/M10187 | HF586369 | 738 | R9 |
|  | *Leishmania naiffi* | MHOM/00/94/CRE58 | HF586374 | 738 | R9 |
|  | *Leishmania siamensis* | / | KC20288 | 738 | R12 |
|  | *Trypanosoma brucei* | / | KP208736 | 738 | R14 |

**References**

[R1] Yang BB, Chen DL, Chen JP, Liao L, Hu XS, Xu JN. Analysis of kinetoplast cytochrome *b* gene of 16 *Leishmania* isolates from different foci of China: different species of *Leishmania* in China and their phylogenetic inference. Parasit Vectors. 2013;6:32.

[R2] Asato Y, Oshiro M, Myint CK, Yamamoto Y, Kato H, Marco JD, et al. Phylogenic analysis of the genus *Leishmania* by cytochrome *b* gene sequencing. Exp Parasitol. 2009;21:352–61.

[R3] Spotin A, Rouhani S, Parvizi P. The associations of *Leishmania* *major* and *Leishmania tropica* aspects by focusing their morphological and molecular features on clinical appearances in Khuzestan Province, Iran. Biomed Res Int. 2014; 2014:913510.

[R4] Luyo-Acero GE, Uezato H, Oshiro M. Sequence variation of the cytochrome *b* gene of various human infecting members of the genus *Leishmania* and their phylogeny. Parasitology. 2004;128:483–91.

[R5] Fotouhi-Ardakani R, Dabiri S, Ajdari S. Assessment of nuclear and mitochondrial genes in precise identification and analysis of genetic polymorphisms for the evaluation of *Leishmania* parasites. Infect Genet Evol. 2016;46:33–41.

[R6] Foulet F, Botterel F, Buffet P, Morizot G, Rivollet D. Detection and identification of *Leishmania* species from clinical specimens by using a real-time PCR assay and sequencing of the cytochrome *b* gene. J Clin Microbiol. 2007;45:2110–5.

[R7] de la Cruz VF, Neckelmann N, Simpson L. Sequences of six genes and several open reading frames in the kinetoplast maxicircle DNA of *Leishmania tarentolae*. J Biol Chem. 1984;259:15136–47.

[R8] Brochu C, Haimeur A, Ouellette M. The heat shock protein HSP70 and heat shock cognate protein HSC70 contribute to antimony tolerance in the protozoan parasite *Leishmania*. Cell Stress Chaperones. 2004;9:294–303.

[R9] van der Auwera G, Maes I, de Doncker S, Ravel C. Heat-shock protein 70 gene sequencing for *Leishmania* species typing in European tropical infectious disease clinics. Euro Surveill. 2013;18:20543.

[R10]Fraga J, Montalvo AM, de Doncker S. Phylogeny of *Leishmania* species based on the heat-shock protein 70 gene. Infect Genet Evol. 2010;10:238–45.

[R11]Bock JH, Langer PJ. Sequence and genomic organization of the hsp70 genes of *Leishmania* *amazonensis*. Mol Biochem Parasitol. 1993;62:187–97.

[R12]Leelayoova S, Siripattanapipong S, Hitakarun A, Kato H, Tan-ariya P, Siriyasatien P, et al. Multilocus characterization and phylogenetic analysis of *Leishmania siamensis* isolated from autochthonous visceral leishmaniasis cases, southern Thailand. BMC Microbiol. 2013;13:60.

[R13]Johnson BJ, Hill GC, Donelson JE. The maxicircle of *Trypanosoma brucei* kinetoplast DNA encodes apocytochrome b. Mol Biochem Parasitol. 1984;13:135–46.

[R14]Fraga J, Fernandez-Calienes A, Montalvo AM, Maes I, Deborggraeve S, Buscher P, et al. Phylogenetic analysis of the *Trypanosoma* genus based on the heat-shock protein 70 gene. Infect Genet Evol. 2016;43:165–72.
